# Non-parent of Origin Expression of Numerous Effector Genes Indicates a Role of Gene Regulation in Host Adaption of the Hybrid Triticale Powdery Mildew Pathogen

**DOI:** 10.3389/fpls.2018.00049

**Published:** 2018-01-30

**Authors:** Coraline R. Praz, Fabrizio Menardo, Mark D. Robinson, Marion C. Müller, Thomas Wicker, Salim Bourras, Beat Keller

**Affiliations:** ^1^Department of Plant and Microbial Biology, University of Zürich, Zürich, Switzerland; ^2^Department of Molecular Life Sciences and SIB Swiss Institute of Bioinformatics, University of Zürich, Zürich, Switzerland

**Keywords:** *Blumeria graminis*, effectors, host adaptation, hybridization, plant pathogenic fungi, powdery mildew, RNA-Seq

## Abstract

Powdery mildew is an important disease of cereals. It is caused by one species, *Blumeria graminis*, which is divided into *formae speciales* each of which is highly specialized to one host. Recently, a new form capable of growing on triticale (*B.g. triticale*) has emerged through hybridization between wheat and rye mildews (*B.g. tritici* and *B.g. secalis*, respectively). In this work, we used RNA sequencing to study the molecular basis of host adaptation in *B.g. triticale*. We analyzed gene expression in three *B.g. tritici* isolates, two *B.g. secalis* isolates and two *B.g. triticale* isolates and identified a core set of putative effector genes that are highly expressed in all *formae speciales*. We also found that the genes differentially expressed between isolates of the same form as well as between different *formae speciales* were enriched in putative effectors. Their coding genes belong to several families including some which contain known members of mildew avirulence (*Avr*) and suppressor (*Svr*) genes. Based on these findings we propose that effectors play an important role in host adaptation that is mechanistically based on *Avr-Resistance gene-Svr* interactions. We also found that gene expression in the *B.g. triticale* hybrid is mostly conserved with the parent-of-origin, but some genes inherited from *B.g. tritici* showed a *B.g. secalis*-like expression. Finally, we identified 11 unambiguous cases of putative effector genes with hybrid-specific, non-parent of origin gene expression, and we propose that they are possible determinants of host specialization in triticale mildew. These data suggest that altered expression of multiple effector genes, in particular *Avr* and *Svr* related factors, might play a role in mildew host adaptation based on hybridization.

## Introduction

Crops are threatened by rapidly evolving pathogen populations, resulting in economically important yield losses and requiring continuous breeding for new resistant cultivars (Oerke, 2006). In cereals, plant pathogenic fungi are considered the most important cause of disease and pathogens such as powdery mildew and leaf rust can cause up to 20 and 50% of yield losses in wheat, respectively (Conner et al., 2003; Huerta-Espino et al., 2011; Dean et al., 2012). These two pathogens are obligate biotrophs that require living host cells to complete their life cycle. To do so, a very close interaction with the host is established through the formation of a haustorium. This invasive feeding structure allows the fungus to take up nutrients and deliver effector proteins into the plant cell. Biotrophic fungi encode for a remarkably large repertoire of so called effectors, as compared to hemi-biotrophic or necrotrophic fungal plant pathogens (Lo Presti et al., 2015). For instance, over 1,000 putative effectors have been identified in the genome of rust fungi from the genus *Melampsora* (*Basidiomycetes*) (Hacquard et al., 2012; Saunders et al., 2012) and over 500 in cereal powdery mildews (Pedersen et al., 2012; Praz et al., 2017).

Such large effector repertoires with possibly high levels of functional redundancy are hypothesized to provide a selective advantage to increase fitness of the pathogen (Birch et al., 2008). However, effectors that are recognized by plant resistance proteins, so called avirulence proteins, result in restriction of growth of the pathogen to specific host species or cultivars. Only few avirulence genes have been cloned so far from obligate biotrophic fungi and the majority was isolated from the flax rust fungus *Melampsora lini* (Dodds et al., 2006; Ravensdale et al., 2011). In cereal powdery mildews, four avirulence effector genes were identified so far: two in barley powdery mildew and two in wheat powdery mildew. In barley powdery mildew, *Avr*_*a1*_ *and Avr*_*a13*_ are recognized by *Mla1 and Mla13*, respectively, two alleles of the barley *Mla* resistance gene (Lu et al., 2016). In wheat powdery mildew, *AvrPm3*^*a2*/*f2*^ is recognized by *Pm3a* and *Pm3f*, two alleles of the wheat *Pm3* resistance gene, while *AvrPm2* is recognized by *Pm2* (Bourras et al., 2015; Praz et al., 2017). In addition to putative effectors acting as canonical *Avr* genes, wheat powdery mildew also encodes for a suppressor of avirulence, *SvrPm3*^*a1*/*f1*^, a putative effector gene capable of suppressing the *AvrPm3-Pm3* mediated race-specific resistance (Bourras et al., 2015, 2016; Parlange et al., 2015). It was also shown that mildew *Avr* and *Svr* genes are highly upregulated at 2 days post infection, which is consistent with the formation of the haustorial feeding structure, a landmark for successful host colonization.

Despite the identification of several effectors acting as avirulence factors in biotrophic plant pathogens, the primary biological and biochemical functions of these proteins and their role in fungal virulence remain largely unknown. It was shown in different plant pathogenic fungi that effectors are involved in virulence by either suppressing plant immunity or by avoiding recognition by the plant. In *Ustilago maydis*, the effector Pit2 inhibits a set of cysteine proteases that promote salicylic acid-associated plant defenses. Pep1, another effector of the same pathogen, suppresses plant immunity by inhibiting a component of the ROS-generating system (Hemetsberger et al., 2012). In *Cladosporium fulvum*, Ecp6 sequesters oligosaccharides from invading hyphae to prevent elicitation of host immunity (de Jonge et al., 2010). In barley powdery mildew, host induced gene silencing (HIGS) was used to downregulate the expression of effector genes and revealed a functional role in fungal virulence and haustoria formation of 10 effectors [BEC1005, BEC1011, BEC1016, BEC1018, BEC1019, BEC1038, BEC1040, and BEC1054 (Pliego et al., 2013); CSEP0105 and CSEP0162 (Ahmed et al., 2015), and CSEP0055 (Zhang et al., 2012)]. BEC1054 was later shown to target several barley proteins including a pathogenesis-related-5 protein isoform, suggesting a role in suppressing pathogen-associated molecular pattern (PAMP)-triggered immunity (PTI) (Pennington et al., 2016a); CSEP0105 seems to promote fungal virulence by interfering with a barley small heat shock protein (Ahmed et al., 2015) and CSEP0055 contributes to the aggressiveness of the fungus, especially at sites of secondary penetration, and interacts with the barley pathogenesis-related protein PR17c (Zhang et al., 2012). Thus, although the molecular function of most of the effectors of biotrophic fungi remains unknown, there is some evidence that effectors are relevant for virulence of biotrophic fungi, and this also in cereal powdery mildews.

Genome-wide transcriptomic studies have been increasingly used to study the genes involved in fungal virulence and host-pathogen interactions, in particular putative effector genes. Several transcriptomic studies in biotrophic fungal pathogens such as *Puccinia striiformis*, as well as hemibiotrophs such as *Magnaporthe grisea, Zymoseptoria tritici*, and *Leptosphaeria maculans* all found strong upregulation of putative effector genes during host infection, thus substantiating their role in host-pathogen interactions (Kawahara et al., 2012; Garnica et al., 2013; Hacquard et al., 2013; Sonah et al., 2016). It was also shown that effector gene expression in plant pathogenic fungi occurs in different waves during host invasion, suggesting stage-specific regulation of different subsets of the effector repertoire (Cantu et al., 2013; Hacquard et al., 2013; Rudd et al., 2015; Palma-Guerrero et al., 2016; Sonah et al., 2016). In *Leptosphaeria maculans*, stage-specific expression of different effector waves was identified in transcriptomic studies comparing cotyledon and stem colonization of oilseed rape by the pathogen (Sonah et al., 2016). Finally, studies of quantitative differences in gene expression between pathogen races and lineages revealed the existence of both isolate and lineage-specific patterns of gene expression (Duplessis et al., 2011; Palma-Guerrero et al., 2017). In particular, Duplessis and colleagues found that most of the effectors upregulated in wheat stem rust and poplar leaf rust are lineage-specific, suggesting possible roles in host adaptation (Duplessis et al., 2011).

Cereal powdery mildew diseases are caused by one species, *Blumeria graminis* (*B.g*.), which is divided in subspecies called *formae speciales* (*ff.spp*.), each of which is adapted to a specific host species (Troch et al., 2014b). Five mildew *formae speciales* grow on cultivated cereals: *B.g. tritici* on wheat, *B.g. hordei* on barley, *B.g. secalis* on rye, *B.g. avenae* on oat, and *B.g. triticale* on triticale and wheat (Troch et al., 2014b). Of these, triticale powdery mildew is the most recently described form of mildew that has emerged in Europe in the early 2000s (Troch et al., 2012, 2014a). *B.g. triticale* arose from a hybridization between *B.g. tritici* and *B.g. secalis* followed by backcrosses in *B.g. tritici* and leading to a hybrid genome showing a mosaic structure of segments of the wheat and rye mildew donors (Menardo et al., 2016). In particular, it was shown that the genome composition and evolution of *B.g. triticale* is reminiscent of that of the host, triticale, which is also a recent artificial hybrid of wheat and rye, introduced as an agricultural crop in the twentieth century. It is not known how gene expression in the hybrid mildew is affected and whether parental expression patterns are maintained. In particular, it is not known if there are genes with hybrid-specific regulation in the triticale mildew, and if such regulation can contribute to host-adaptation at the transcriptional level.

Here we study gene expression levels in the hybrid triticale mildew and in the parental wheat and rye mildew genome donors. We identify a core set of genes that are highly expressed irrespective of the *forma specialis*, and show they are highly enriched in candidate effectors. We also identify differentially expressed genes between isolates and between *formae speciales*, and show they are also enriched in candidate effectors. We show that gene regulation in the hybrid is biased toward a *B.g. secalis*-like expression, and identify putative effectors with non-parent of origin gene expression. Finally, we propose that transcriptional regulation represents an additional level of polymorphisms controlling host adaptation where effectors can be activated to suppress resistance or inactivated to escape recognition.

## Materials and methods

### Fungal isolates, plant growth conditions, inoculation, and tissue harvest

The isolates of *B.g. tritici* (96224, JIW2 and 94202) were propagated on *Triticum aestivum* cultivar “Chinese Spring” and the *B.g. secalis* isolates (S-1391 and S.1459) on cultivar “Matador” of *Secale cereale*. Ten days old detached leaf segments were infected with fresh spores and the infected leaf segments were kept on benzimidazole agar plates at 20°C and 70% humidity with a 16 h light/8 h dark cycle (Parlange et al., 2011). The *B.g. triticale* isolates THUN-12 and T3-8 were maintained the same way on triticale cultivar Timbo and were previously described in Menardo et al. (2016).

### RNA extraction and sequencing

Each pathogen-host infection combination was replicated three times and leaf segments of each of the replicates were collected at 2 days after infection. The leaf segments were directly frozen in liquid nitrogen and total RNA was extracted using the miRNeasy mini kit from Qiagen according to the manufacturer's instructions (Qiagen). RNA quality was checked by gel electrophoresis and also based on the 260:280 ratios measured with a spectrophotometer (Nanodrop, Thermo Fisher Scientific, Waltham, MA, USA). 100-bp single end stranded libraries were created for the *B.g. tritici* isolates and 125-bp single-end stranded libraries for the *B.g. secalis* isolates. All libraries were sequenced with the Illumina HiSeq platform at the Functional Genomics Center Zürich (http://www.fgcz.ch/) (Supplementary Table 1). The *B.g. triticale*—triticale libraries were produced as previously described in Menardo et al. (2016, Supplementary Table 1).

### Reference genome improvement and annotation

Illumina sequences of the reference isolate 96224 were obtained and aligned to the reference genome (Wicker et al., 2013) using CLC Genomics Workbench version 7.5 with automatic word and bubble size. The Extract Consensus Sequence function from CLC was used to correct the reference genome with a minimum coverage of 10. Four loci were recently re-sequenced in different *Avr* gene mapping projects: the loci containing *AvrPm3*^*a*2/*f*2^ (Accession number KT714072), *SvrPm3*^*a*1/*f*1^ (available upon request) and *AvrPm2* (Accession number KX765276) (Bourras et al., 2015; Parlange et al., 2015; Praz et al., 2017) and an additional locus obtained from BAC sequencing (unpublished data, available upon request). All sequences were integrated into an updated version of the genome version. Finally, the annotation obtained by Praz and colleagues was transferred to this new reference sequence (Praz et al., 2017). The mildew genome sequence, the corresponding GFF annotation, and all gene sequences (CDS) used in this study are available under the accession number PRJEB23548.

### Mapping

Prior to the mappings, a genome index was generated for the *B.g. tritici* reference genome including the annotation file (CDS) with the following command: STAR –runMode genomeGenerate –genomeFastaFiles –sjdbGTFtagExonParentTranscript –sjdbOverhang 100.

Quality control was done using FastQC (https://www.bioinformatics.babraham.ac.uk/projects/fastqc/) on all RNA-seq libraries (see Supplementary Figure 1). We tested four different mapping parameters using the sequence aligner software STAR (Dobin et al., 2013) with all libraries and finally the libraries were mapped on the improved *B.g. tritici* reference genome using STAR with the following command: STAR –outFilterMultiMapNmax 1 – outFilterMismatchNoverLmax 0.01 – alignIntronMax 500. All genomic sequencing read libraries (see Supplementary Table 2) were also mapped using STAR (Dobin et al., 2013) using the same parameters.

### Identification of presence/absence gene polymorphism

We used featureCount 1.4.6 to obtain read count value for genomic coverage of the 7,138 annotated mildew genes (Liao et al., 2014) using the mapping obtained by aligning the genomic reads of the isolates on the reference genome with STAR. Read count normalizations were performed based on library size and gene length. A coverage value was obtained for every gene in every isolate and, after manual curation, genes with a coverage smaller than 5 were considered as missing in the corresponding isolate (Supplementary Figure 2, Supplementary Text 1).

### Expression analyses

Read counts were determined from the mapping files with featureCounts 1.4.6 (Liao et al., 2014) using default parameters and the –M option that specifies that multi-mapping reads should be counted. Statistical analysis was done with the R package edgeR and genes were tested for differential expression with a general linear model and tagwise estimation of dispersion (Robinson et al., 2010). Normalization for RNA composition was performed in edgeR using the command calcNormFactors(). A gene was considered as expressed if the cpm (count per million) was >10 in at least three of the 21 replicates. We used three different designs for the comparison between isolates, *formae speciales* and specific analysis of expression in the hybrid, and estimated dispersion using the following functions: estimateGLMCommonDisp(), estimateGLMTrendedDisp(), and estimateGLMTagwiseDisp(). We fitted a negative binomial generalized log-linear model to the read count for each gene using the glmFit() function and tested for differential expression with the glmLRT() function with different contrasts for pairwise comparisons. The R Script where all these parameters are detailed is available as Supplementary Data Sheet 1.

A gene was considered differentially expressed if it had a log2FC > |1.5| with an adjusted *p*-value (FDR) < 0.01. In addition we also performed the same analyses with different thresholds: log2FC < |1|, log2FC < |2|, log2FC < |2.5|. In the study of gene expression conservation in the hybrid triticale mildew, the expression of a gene in a *B.g. triticale* isolate was considered similar to *B.g. tritici* or *B.g. secalis* when log2FC < |1.5|. Pairwise comparisons were used to compare isolates and *formae speciales*. For the expression in the hybrid, four pairwise comparisons were done: T3-8 vs. *B.g. tritici*, T3-8 vs. *B.g. secalis*, THUN-12 vs. *B.g. tritici* and THUN-12 vs. *B.g. secalis*. The Venn diagramms were produced using EulerAPE (Micallef and Rodgers, 2014).

We have also compared our mapping and read count strategy with the RNA-Seq analysis pipeline “Salmon” (Patro et al., 2017), using standard parameters. Correlation between the two strategies was assessed using the “lm” function form the R package “stats” for fitting linear models.

### Gene enrichment and gene ontology analyzes

The list of candidate effector genes as well as their classification in families used in this study was obtained from Praz et al. (2017). Sequences of those 595 putative effectors are available as Supplementary Data Sheet 3 and the list of putative effector families as Supplementary Data Sheet 4. We considered all putative effectors, non-effector secreted proteins (identified with SignalP, Nielsen et al., 1997; Petersen et al., 2011) and putative proteins involved in the secretory pathway (identified with TargetP, Emanuelsson et al., 2000) as the secretome. Enrichment analyses were performed with R using Fisher's exact test and results with a *p*-value smaller than 0.01 were considered significant.

To perform GO enrichment analyses, we used Blast2Go (Götz et al., 2008). We first performed a Blastx search against the NCBI non-redundant (nr) database (https://www.ncbi.nlm.nih.gov). Standard parameters were used to filter the output, and hits were considered significant if the blast *p*-value was smaller than 10e-8. Only 20 hits per gene sequence were kept. These results were imported into the Blast2go interface and subsequent analyses were done as described in the Blast2go manual (https://www.blast2go.com/images/b2g_pdfs/b2g_user_manual.pdf). First an InterProScan search was performed and then the GO terms obtained were added to the annotation. Later the GO terms were mapped and the annotations of “molecular function,” “biological process,” and “cellular component” were performed. Finally enrichment analyses were done for the different sets of genes identified using the “Enrichment Analysis” function of Blast2Go. This function uses the Fisher's Exact Test and corrects for multiple testing.

In addition we performed Blast searches against the conserved domain database (CDD) (https://www.ncbi.nlm.nih.gov/Structure/cdd/cdd.shtml) using standard parameters, with the protein sequences of the top5% genes and the DE genes and manually analyzed the data.

### Phylogeny analysis

We used GMAP (version 2016-05-01-1, Wu and Watanabe, 2005) to retrieve the 257 genes in the assembly of 14 *B.g. tritici* isolates, 5 *B.g. secalis* isolates, the two *B.g. triticale* isolates of interest (Menardo et al., 2016), and in *B.g. hordei* genome using the sequences of the *B.g. tritici* reference isolate 96224 as template. We then aligned these genes using muscle (v3.8.31, Edgar 2004) and maximum likelihood trees were inferred with RAXML 8.2.8 (Stamatakis, 2014) using a GTR GAMMA model (Yang, 1993, 1994), and bootstrap support was computed using 10,000 replications. We manually inspected the trees to determine if the gene in all *B.g. tritici* isolates and all *B.g. secalis* isolates cluster in two different clades thus allowing possible separation of sequence origin. *B.g. triticale* haplotypes clustering in a *B.g. tritici* clade were considered as having a coding sequence originating from *B.g. tritici*, and *B.g. triticale* haplotypes clustering in a *B.g. secalis* clade were considered as having a coding sequence originating from *B.g. secalis*.

We defined the promoter as the 1kb region upstream of the gene start. The consensus sequence for the promoter regions was retrieved by mapping of the genomic reads of the *B.g. tritici, B.g. secalis* and the two *B.g. triticale* isolates T3-8 and THUN-12 on the reference genome. We could not retrieve the promoter sequence in *B.g hordei* because mapping of *B.g. hordei* reads on the *B.g. tritici* reference genome is not possible since the two genomes are highly divergent. The consensus sequences were called using bcftools consensus function after selecting only heterozygous SNPs and position with a minimum coverage of 10. These sequences were then aligned using muscle (v3.8.31, Edgar, 2004) and maximum likelihood trees were inferred with RAXML 8.2.8 (Wu and Watanabe, 2005; Stamatakis, 2014) using a GTR GAMMA model (Yang, 1993, 1994), and bootstrap support was computed using 10,000 replications. The trees were manually inspected to define if the promoter sequence in the two *B.g. triticale* isolates cluster in a *B.g. tritici*-clade or in a *B.g. secalis*-clade using the same approach as for the gene sequence.

## Results

### Transcriptomes of wheat, rye, and triticale mildews are distinct

**Figure 1:**
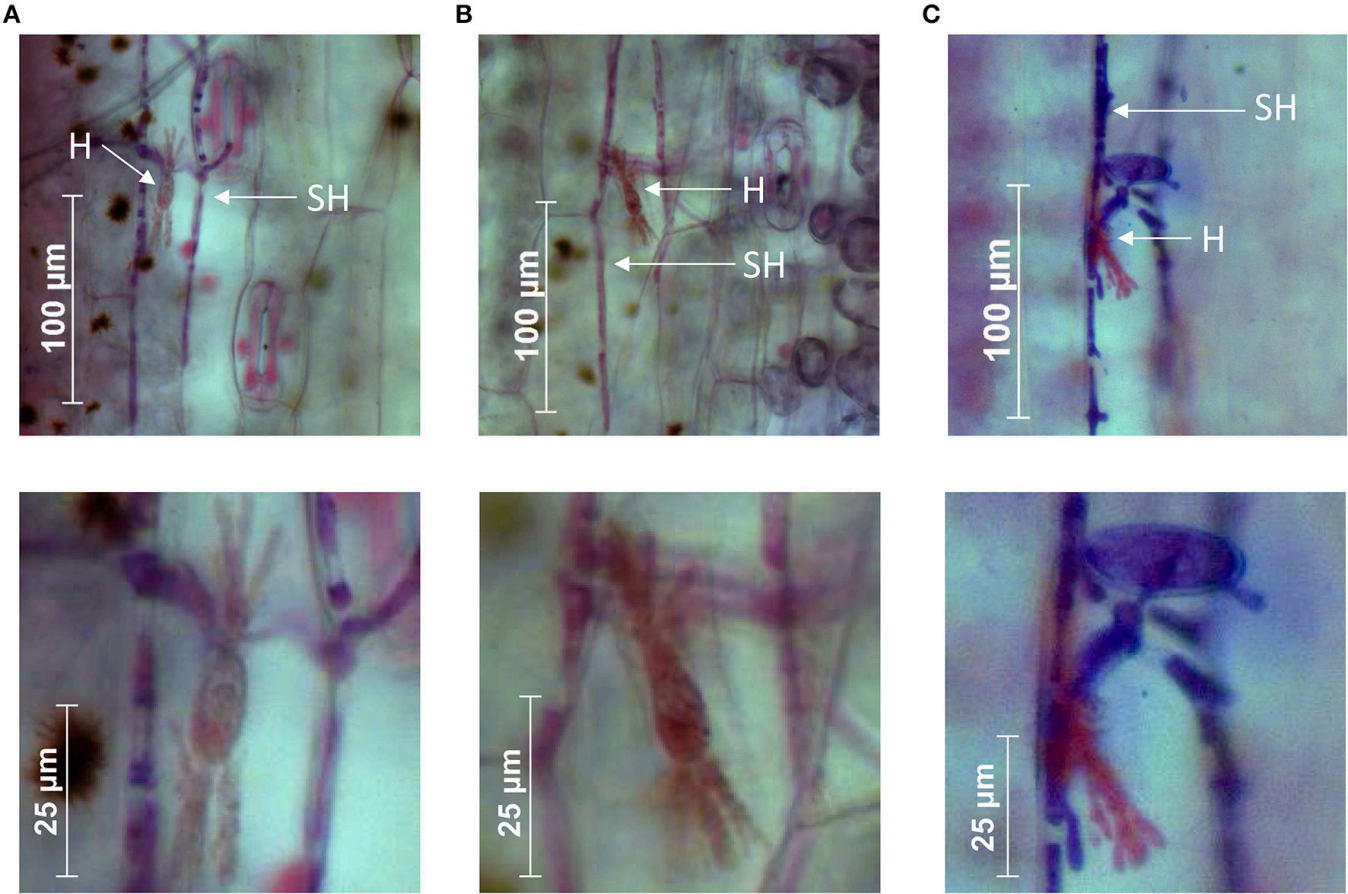
Micrographs of wheat, rye, and triticale powdery mildew growing on their respective susceptible hosts at 2 dpi. **(A)** *B.g. tritici* isolate 96224 on the wheat cultivar “Chinese Spring.” **(B)** *B.g. secalis* isolate S-1391 on the rye cultivar “Matador.” **(C)** *B.g. triticale* isolate THUN-12 on the triticale cultivar “Timbo.” Haustoria are indicated with “H” and secondary hyphae with “SH.” Lower panels show magnifications from the upper panels depicting fully formed haustoria.

We studied gene expression in three isolates of wheat mildew *B.g. tritici* (96224 and 94202 originating from Switzerland and JIW2 from the United Kingdom), two of rye mildew *B.g. secalis* (S-1391 and S-1459 from Switzerland) and two of triticale mildew *B.g. triticale* (T3-8 from Switzerland and THUN-12 from Poland). All isolates were grown on their susceptible hosts (wheat, rye, and triticale, respectively) and samples for RNAseq were collected at 2 days post infection (dpi). At this specific stage of the infection, haustoria were formed, indicating successful plant colonization (Figure 1). RNA from three independent biological samples was extracted from infected leaves and sequenced. The RNAseq data of *B.g. triticale* on triticale were obtained from a previous study (Menardo et al., 2016; see section Materials and Methods).

We first tested four sets of mapping parameters using STAR (Dobin et al., 2013) allowing different thresholds for mismatches and multimappers (see Supplementary Table 3). We found that the percentage of mapped reads was very similar irrespective of the mapping strategy, and we therefore decided to use stringent parameters allowing 1% mismatch and one multimapper. We then used featureCounts to obtain counts values for each replicate. To further validate our mapping strategy, we compared the counts obtained with STAR and featureCounts to those obtained with a recently described method, Salmon (Patro et al., 2017). We found 99.9% correlation between the counts thus demonstrating that our mapping strategy is appropriate (see Supplementary Figure 3).

**Figure 2:**
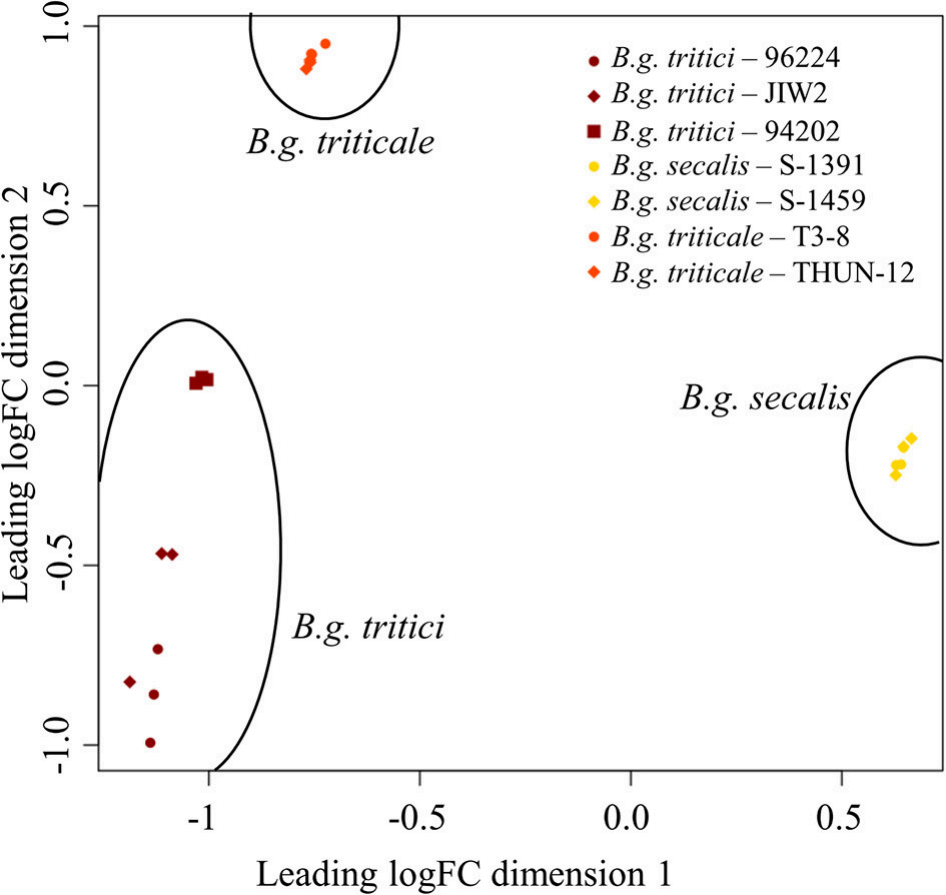
Multidimensional scaling plot of RNAsequencing data. Multidimensional scaling plot representing the 21 RNA-seq libraries. Colors represent the different *formae speciales* and different dots/shapes represent individual isolates. Dimension 1 and dimension 2 separate the libraries based on the expression values of 6,203 genes prior to differential gene expression analysis. Dimension 1 separates the *formae speciales* and shows that the expression of the *B.g. triticale* isolates is closer to the one of *B.g. tritici* than the one of *B.g. secalis*. Dimension 2 separates the isolates of *B.g. tritici*.

With the chosen mapping parameters, we could align 3.7–8.8% of the total reads to the *B.g. tritici* reference genome (Supplementary Table 1), and found that 6,203 out of 7,138 annotated genes were expressed in at least one isolate (see section Materials and Methods). Multidimensional scaling analysis performed on the expression data of those 6,203 expressed genes showed that the different *formae speciales* can be grouped in three distinct clusters, indicating significant differences in gene regulation between wheat, rye, and triticale mildews (Figure 2). This result also shows that the three *formae speciales* can be clearly distinguished based on our whole transcriptome data.

### Highly expressed genes are common to all *Formae speciales* and enriched in candidate effector genes

**Figure 3:**
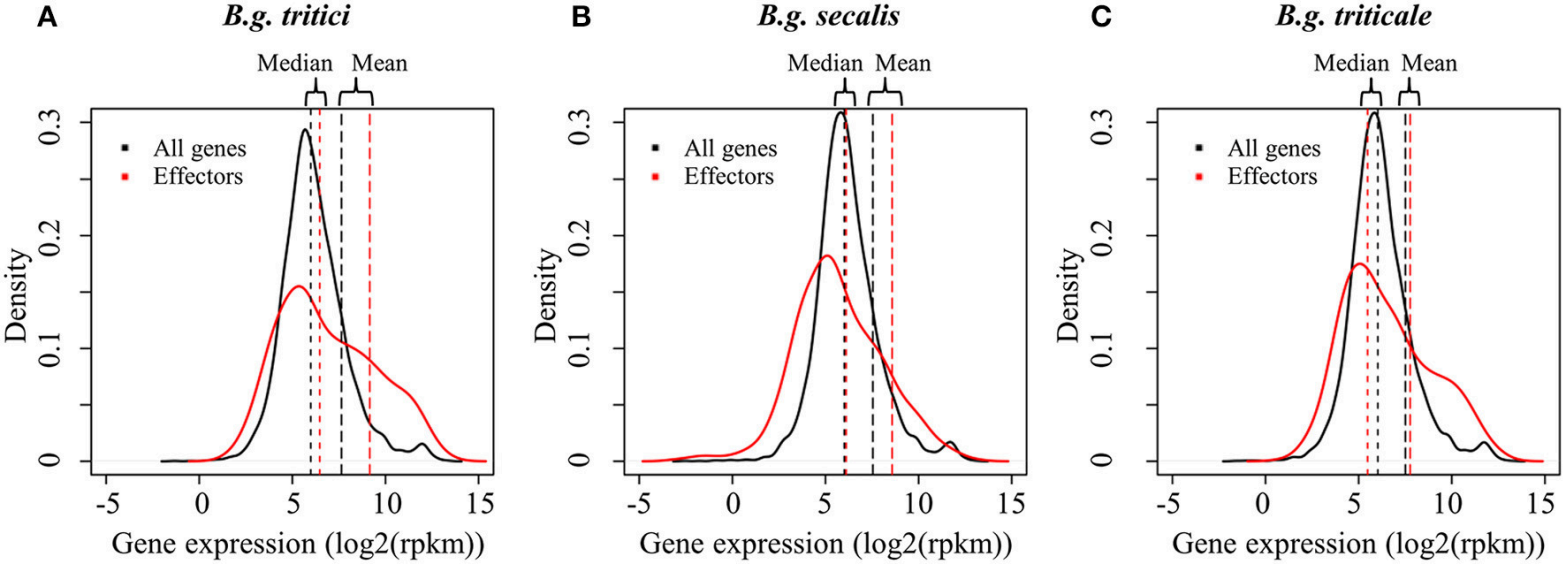
Distribution of gene expression levels in wheat, rye, and triticale mildews. Gene expression distributions in *B.g. tritici* **(A)**, *B.g. secalis* **(B)**, and *B.g. triticale* **(C)** are depicted with black plain lines for all genes and red plain lines for putative effector The distribution of the log2 (rpkm) values of mean gene expression of all isolates and all replicates is plotted. Mean expression and median expression are indicated as vertical lines in black for all genes and in red for effectors genes. All plots show that there is a higher proportion of effectors in the highly expressed genes as compared to non-effector genes.

To quantify and compare gene expression levels in the studied isolates we used a pipeline for RNAseq studies based on R and the edgeR package (R Development Core Team, 2005; Robinson et al., 2010). We considered the genes encoding candidate effectors (595 genes) (Praz et al., 2017) as a separate functional group likely involved in fungal virulence, and containing potential targets of the plant immune system. Using a genome-wide comparison of gene expression levels, we found that the mean expression of candidate effector genes was significantly higher than the gene expression average, irrespective of the mildew isolate or the *forma specialis* (Table 1, Figure 3).

**Table 1:** Gene expression characteristics for all isolates of the three *formae speciales*.

***Forma specialis***	**Isolate**	**5% quantile**		**Median**		**Mean**		**95% quantile**	
		**Non-effectors**	**Effectors**	**Non-effectors**	**Effectors**	**Non-effectors**	**Effectors**	**Non-effectors**	**Effectors**
*B.g. tritici*	96224	12.7	7.8	62.9	83.1	203.1	615.1	624.2	3, 394.8
	JIW2	14.9	8.1	63.4	79.3	186.2	531.1	580.0	2, 835.0
	94202	12.1	10.5	63.2	88.5	207.8	572.7	642.1	3, 029.2
*B.g. secalis*	S-1391	13.7	4.3	64.3	44.3	188.5	230.6	551.3	996.5
	S-1459	14.9	5.2	65.5	44.8	176.7	208.4	536.0	918.9
*B.g. triticale*	T3-8	14.4	7.9	64.9	65.4	186	364.2	564.5	1, 820.4
	THUN-12	14.2	9.0	64.2	74.2	187.1	406.6	568.6	2, 074.2

*The 5% quantile, median, mean, and 95% quantile values are provided for non-effector genes (abbreviated “Non-effectors”) and for candidate effector genes. All values are in reads per kilo base pair per million reads (rpkm). The candidate effector genes are in general more expressed than the other genes*.

**Figure 4:**
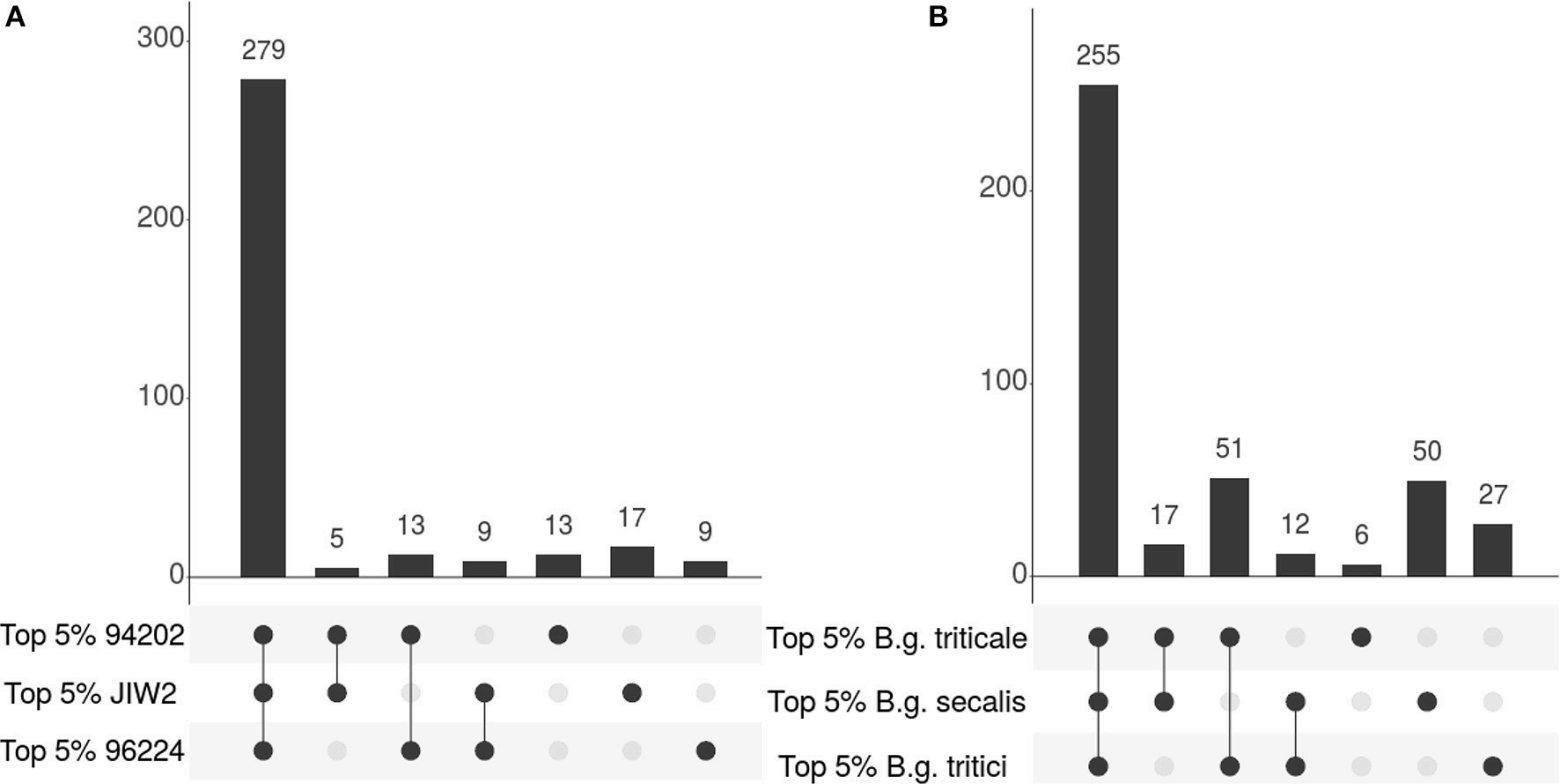
Intersections plots showing the overlaps of different sets of highly expressed genes. Histograms represent the number of genes contained in each group present in the overlaps indicated in the lower panel. Highly expressed genes are defined as the top 5% with the highest expression values (see text). **(A)** Comparison of the three *B.g. tritici* isolates. The first bar represents highly expressed genes common to three isolates, the following three bars represent those common to two isolates and the last three bars those found only in one isolate. **(B)** Comparison of the three *formae speciales*. The first bar represents the core highly expressed (HE) genes common to three *formae speciales*, the following three bars represent those common to two *formae speciales* and the last three bars those found only in one *forma specialis*.

Then, to study the nature of highly expressed genes in more detail, we selected the 5% most highly expressed ones (top 5%) in each isolate. In wheat mildew, we found that 279 out of the top 5% 345 highly expressed genes are common to all three isolates (81%, Figure 4A). Similarly, when comparing across all isolates of wheat, rye and triticale mildew, we found that 255 out of the 418 top 5% genes are common to all *formae speciales* (62.5%, Figure 4B). We will hereafter refer to those 418 genes that are in the top 5% in at least one of the studied isolates as the “most HE genes,” and refer to the subset of 255 genes that are highly expressed in all three *ff.spp*. as the “core HE genes.”

To further analyze the most HE genes for specific gene composition or predicted protein function, we first used gene ontology (GO) annotations for “molecular function,” “cellular component,” and “biological process” and compared the results to genome-wide GO annotations (see Supplementary Data Sheet 2). We found no statistically significant enrichment in any specific GO term in this gene set, and the same was found when extending the analysis to a sample including the top 10% most highly expressed genes. We then performed blast searches against protein conserved domain databases (https://www.ncbi.nlm.nih.gov/Structure/cdd/cdd.shtml) and found a high proportion of proteins involved in basal cellular processes (e.g., ribosomal proteins), indicating a massive investment of the fungus in basal metabolism and housekeeping functions consistent with invasive fungal growth at 2 dpi (see Supplementary Table 4).

Most importantly, we found a significant enrichment in candidate effector genes (Fisher exact test *p*-value < 0.01) as they account for 28, 24, and 13% of the top 5% in wheat, triticale and rye mildews, whereas they represent 8, 7.8, and 7% of the total expressed genes in the same *formae speciales*, respectively (Table 2). Similarly, when considering the most HE and core HE genes, candidate effectors represent 27.3% (114 genes) and 11.4% (29 genes) of each gene set, respectively. Of these 114 candidate effector genes, (i) fifteen are members of the *AvrPm3*^*a*2/*f*2^ family (Bourras et al., 2015), (ii) seven belong to the *AvrPm2* family which also contains the barley mildew avirulence gene *Avr*_*a*13_, and two functionally characterized mildew effectors BEC1011 and BEC1054 (Pliego et al., 2013; Lu et al., 2016; Pennington et al., 2016a; Praz et al., 2017), (iii) *four* are members of the wheat powdery mildew *SvrPm3*^*a*1/*f*1^ suppressor gene family (Bourras et al., 2015; Parlange et al., 2015), (iv) fourteen are members of the family containing the functionally characterized BEC1016 effector (Pliego et al., 2013), and finally, (v) eight are members of the family containing the functionally characterized BEC1038 effector (Pliego et al., 2013) (see Supplementary Table 5). These results indicate that highly expressed candidate effectors genes are likely to encode for important virulence factors in mildew, including several members of gene families encoding functionally validated *Avr* genes (e.g., *AvrPm3*^*a*2/*f*2^*, AvrPm2*) and suppressors of plant immunity (e.g., BEC1011, BEC1054, *SvrPm3*^*a*1/*f*1^). They also suggest high conservation of the basal mechanisms of host infection in wheat, rye, and triticale powdery mildew, based on highly conserved effector function, and possibly common targets in their respective hosts.

**Table 2:** Proportion of candidate effector genes in the top 10% and top 5% most highly expressed genes in different isolates of the three *formae speciales*.

***Formae specialis***	**Isolate**	**Whole genome^a^**			**Top 10% expressed genes^b^**				**Top 5% expressed genes^c^**			
		**All genes**	**Effectors**	**(%)^d^**	**All genes**	**Effectors**	**(%)^d^**	***p*-value^e^**	**All genes**	**Effectors**	**(%)^d^**	***p*-value^e^**
*B.g. tritici*	96224	7133	595	8.3	620	141	22.7	<2.2e-16	345	95	27.5	<2.2e-16
	JIW2	7114	591	8.3	620	137	22.1	<2.2e-16	345	93	27.0	<2.2e-16
	94202	7120	592	8.3	620	140	22.6	<2.2e-16	345	95	27.5	<2.2e-16
*B.g. secalis*	S-1391	6986	523	7.5	620	70	11.3	0.0023	337	46	13.6	0.0012
	S-1459	6983	522	7.5	620	71	11.5	0.0017	337	43	12.8	0.0050
*B.g. triticale*	T3-8	7094	579	8.2	620	112	18.1	<2.2e-16	340	74	21.8	8.6e-16
	THUN-12	7091	580	8.2	620	119	19.2	<2.2e-16	340	84	24.7	<2.2e-16

a Total number of genes and candidate effectors found in mildew genome.

b Total number of genes and candidate effectors found in the to 10% most highly expressed genes.

c Total number of genes and candidate effectors found in the to 5% most highly expressed genes.

d Percentage of candidate effectors in the considered gene set.

e *P-value of the Fisher's exact test for enrichment in candidate effectors. A gene set is considered enriched in effectors if p-value < 0.01*.

### Differentially expressed genes between isolates are enriched in candidate effectors and are *Forma specialis* specific

In order to distinguish differential expression from simple indel polymorphism, we screened the genomes of the seven isolates for instances of gene presence/absence polymorphism. For this, we used genome sequencing coverage to identify possible cases of gene deletions in the studied isolates (see section Materials and Methods, Supplementary Text 1, Supplementary Figure 2, Supplementary Table 6). In total we found 193 genes deleted in at least one isolate, with most cases of gene deletions in the *B.g. secalis* isolates (see Supplementary Table 6). All downstream analyses of differential gene expression were accordingly curated for indel gene polymorphism based on this list of 193 presence/absence variation.

**Figure 5:**
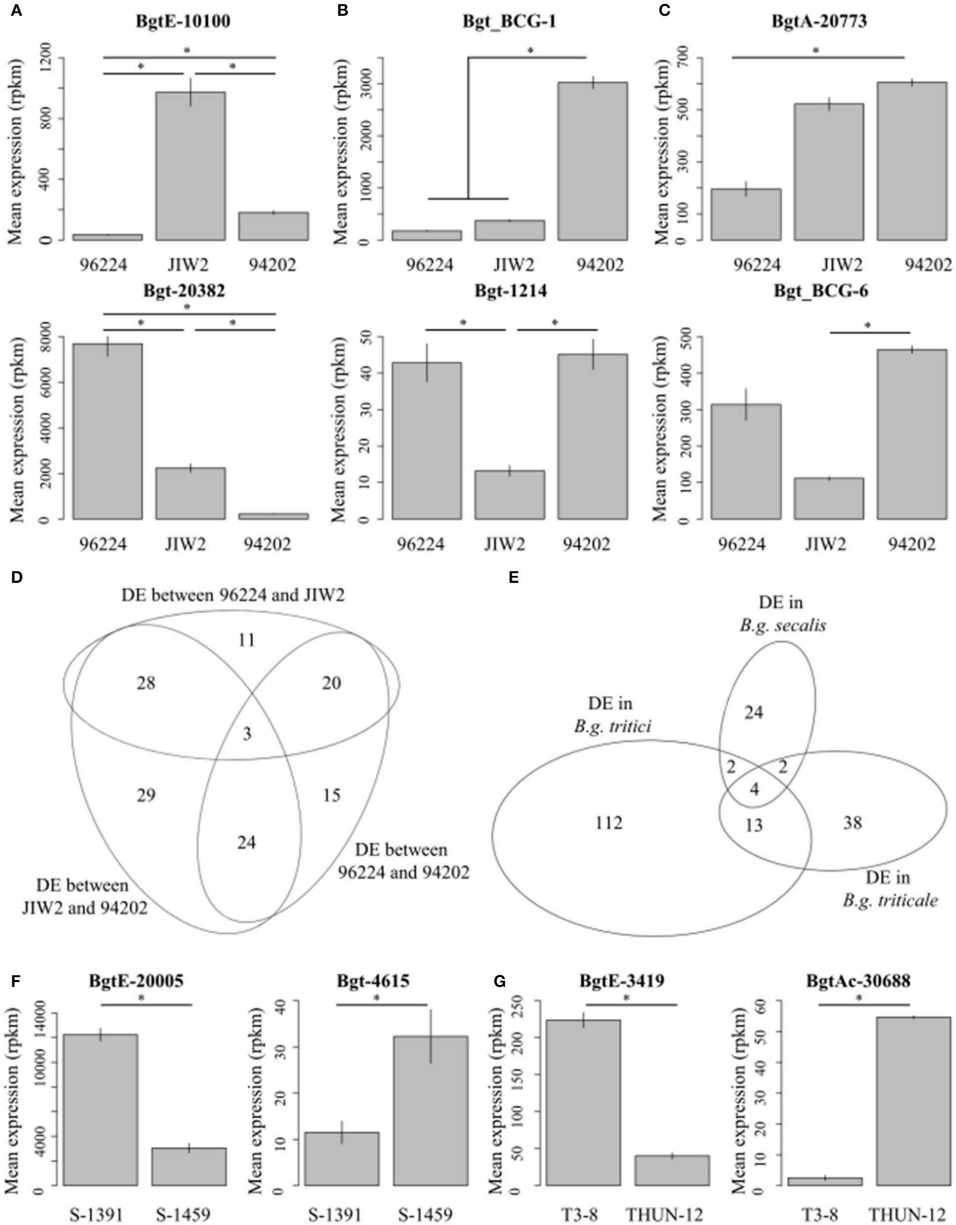
Intra-*forma specialis* gene differential gene expression. Selected examples of differentially expressed genes between isolates of the same *forma specialis* are indicated for *B.g. tritici* **(A–C)**, *B.g. secalis* **(F)**, and *B.g. triticale* **(G)**. ^*^Indicate that the expression of the gene is significantly different between two isolates (comparisons are marked by horizontal lines). **(A)** Shows two example of genes that are differentially expressed between each pair of isolates. **(B)** Shows two examples of genes that are differentially expressed between one isolate and the two others. **(C)** Shows two examples of genes that are differentially expressed between two isolates but show intermediate and non-significantly different expression in the third isolate. Values indicate mean expression from three biological replicates plotted in reads per kilo base pairs per million reads (rpkm). The overlap between differentially expressed genes in the three *B.g. tritici* isolates is depicted as a Venn diagram in **(D)**. Similarly, the overlap between gene differentially expressed between isolates of each *forma specialis* is depicted in **(E)**. The number of overlapping genes is indicated.

We then first compared gene expression between isolates of the same *f.sp*, using a threshold for differential expression of log_2_ fold change (Log_2_FC) > |1.5| and a false discovery rate (FDR) < 0.01 (see section Materials and Methods). In wheat powdery mildew, we performed pairwise comparisons between the three isolates (96224 vs. 94202; 96224 vs. JIW2, and 94202 vs. JIW2), and found 130 differentially expressed (DE) genes (i.e., fulfilling the Log_2_FC and FDR criteria) with a significant enrichment for genes encoding putative effectors (39 genes, 30%) (Table 3). Of these, three genes showed an isolate-specific level of expression (Figures 5A,D) and all three encode for putative effectors: *Bgt-20382* is a member of the *AvrPm3*^*a*2/*f*2^ family, and *BgtE-10100* and *BgtAc-30857* are members of a functionally uncharacterized putative effector family containing 7 members in *B.g. tritici* and 2 in *B.g. hordei*. *Bgt-20382* and *BgtE-10100* are also part of the most HE genes.

**Table 3:** Summary of differentially expressed genes between isolates of the same *forma specialis*.

***Forma specialis***	**Comparison**	**Differentially expressed**			
		**Genes^a^**	**Effectors^b^**	**Effectors (%)^c^**	***p*-value^d^**
*B.g. tritici*	96224 vs. 94202	62	22	35.5	8.3e-10
	JIW2 vs. 94202	84	27	32.1	1.3e-10
	96224 vs. JIW2	62	21	33.9	5.4e-9
*B.g. secalis*	S-1391 vs. S-1459	36	9	25.0	1.7e-3
*B.g. triticale*	T3-8 vs. THUN-12	57	27	47.4	1.9e-15

a Total number of genes differentially expressed between pairs of isolates from the same forma specialis.

b Number of candidate effectors differentially expressed between isolates of the same forma specialis.

c Percentage of candidate effectors among differentially expressed genes.

d *P-value of the Fisher's exact test for enrichment in candidate effectors. A gene set is considered enriched in effectors if p-value < 0.01*.

Seventy-two genes (55.4%) were differentially expressed in one of the isolates as compared to the two others (e.g., *Bgt_BCG-1* and *Bgt-1214*, Figures 5B,D). One prominent example in the latter group of DE genes is *SvrPm3*^*a*1/*f*1^(Syn. *Bgt_BCG-1*) a putative effector gene involved in race specific adaptation of *B.g. tritici* to wheat cultivars containing the *Pm3* resistance gene (Bourras et al., 2015; Parlange et al., 2015). And finally 55 genes (42.3%) were differentially expressed between two isolates, while expression in the third isolate was intermediate and not significantly different from any of the two others (e.g., *BgtA-20773* and *Bgt_BCG-6*; Figures 5C,D).

Similar results were found when comparing gene expression in the two isolates of *B.g. secalis* (36 DE genes), and the two of *B.g. triticale* (57 DE genes), both showing a significant enrichment in DE putative effectors (25 and 47.4%, respectively) (Table 3, Figures 5F,G). We have also applied all DE genes (a total of 195 genes from the three intra-*forma specialis* comparative analyses) to GO annotation and protein domain prediction, and found no statistically significant enrichment for a specific GO category or protein function (see Supplementary Table 7, Supplementary Data Sheet 2). We also found little overlap in DE genes between wheat, rye, and triticale mildews as 89% (174 out of 195) were found to be differentially expressed only between isolates of the same *forma specialis* (Figure 5E). We also performed the same differential expression analyses between isolates using the three additional mapping strategies (Supplementary Table 3), and found very similar results irrespective of mapping stringency (see Supplementary Table 8).

Thus, our results indicate that DE genes between individual isolates are consistently enriched in candidate effectors irrespective of the *forma specialis*. In contrast to the core set of highly expressed genes, we found little overlap between wheat, rye, and triticale mildew based on differentially expressed genes. These results suggest there are distinctly different sets of candidate effector genes that are differentially regulated at early stages of the infection in a *forma specialis*-specific manner.

### Candidate effector genes are responsible for most of the differences in gene expression between *Formae speciales*

**Figure 6:**
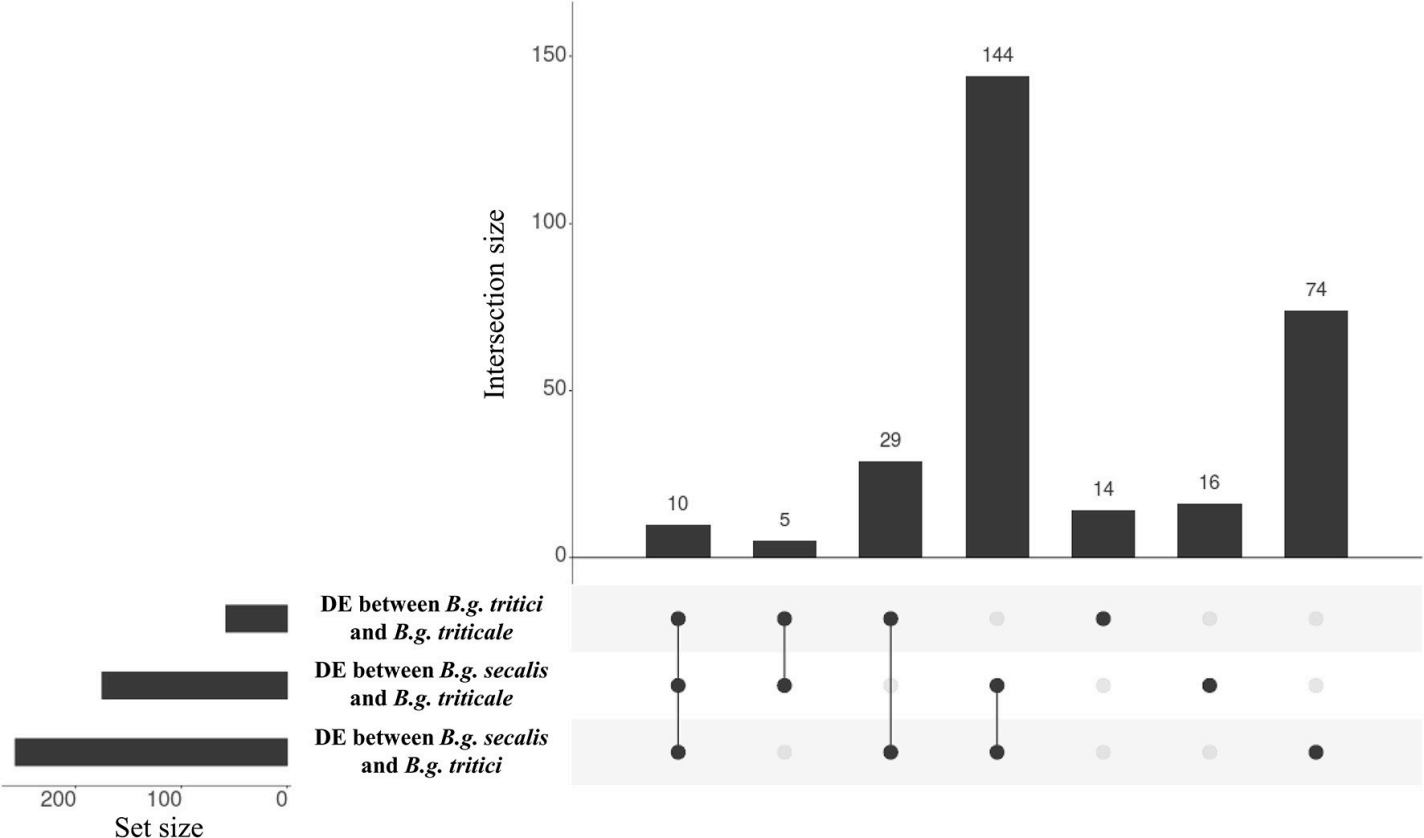
Intersection plots showing the overlap of genes differentially expressed between *formae speciales*. The horizontal histograms represent the number of differentially expressed (DE) genes in each comparison. Gene expression is more similar between *B.g. tritici* and *B.g. triticale* as only 58 genes are differentially expressed between these two *formae speciales*. The vertical histograms represent the number of genes contained in each group present in the overlaps indicated in the lower panel. The first vertical bar represent genes that are differentially expressed between the three *formae speciales*. The three following vertical bars represent genes DE between one *forma specialis* and the two others. Most of the genes are differentially expressed between *B.g. secalis* and the two other *formae speciales*. The last three vertical bars represent genes that are differentially expressed between two *formae speciales*.

**Figure 7:**
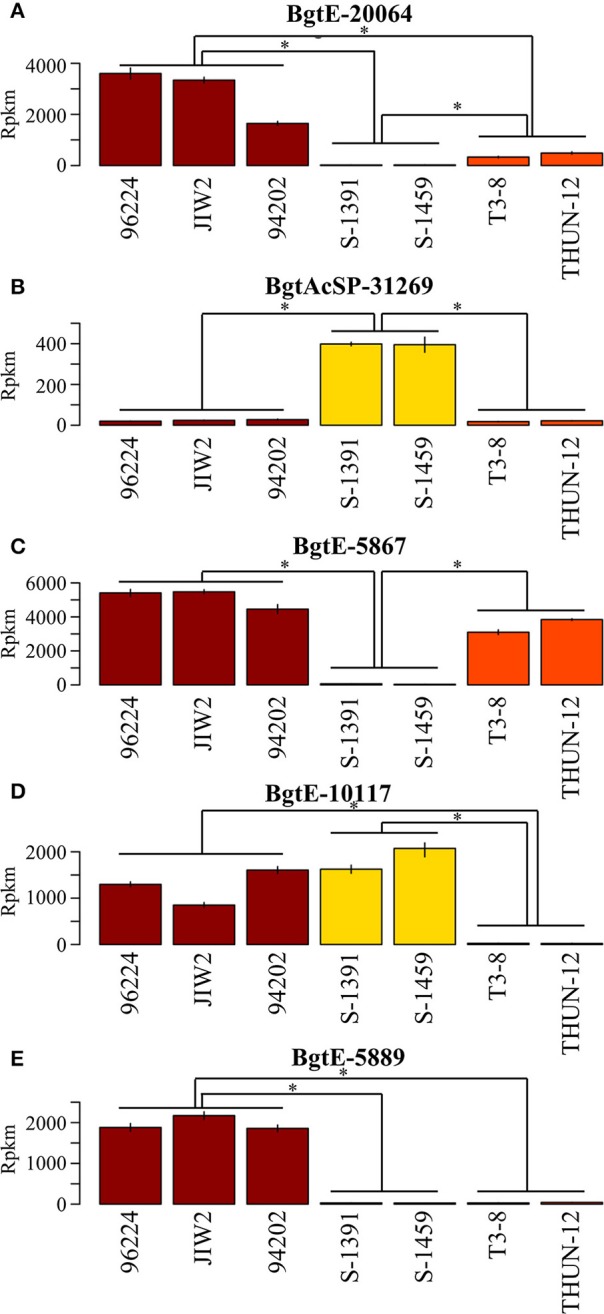
Genes differentially expressed between *formae speciales*. Selected examples of genes differentially expressed between the *B.g. tritici* (red), *B.g. secalis* (yellow) and *B.g. triticale* (orange) *formae speciales*. The mean expression of three biological replicates is plotted in reads per kilo base pairs per million reads (rpkm). ^*^Indicate that the expression of the gene is significantly different between two *formae speciales* (comparisons are marked by horizontal lines). **(A)** *BgtE-20064* is differentially expressed between the three *formae speciales*. **(B,C)** *BgtAcSP-31269* and *BgtE-5867* are differentially expressed between *B.g. secalis* and the two other *formae speciales*. **(D)** *BgtE-10117* is differentially expressed between *B.g. triticale* and the two other *formae speciales*. **(E)** *BgtE-5889* is differentially expressed between *B.g. tritici* and the two other *formae speciales*.

**Figure 8:**
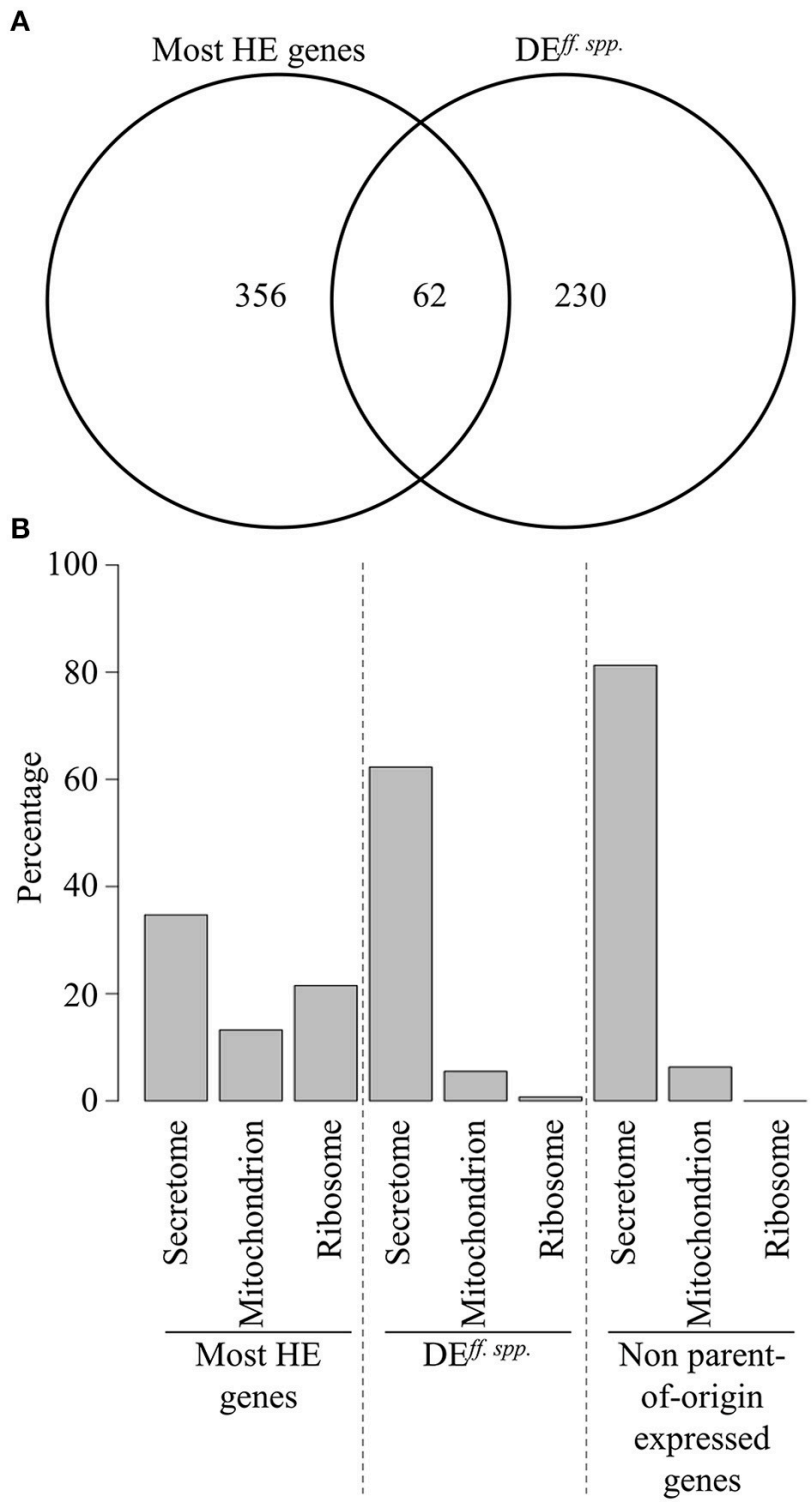
Comparisons of the most highly expressed genes and differentially expressed genes between *formae speciales*. **(A)** Venn diagram showing the overlap between the most highly expressed genes (most HE genes) and the genes differentially expressed between *formae speciales* (${\text{DE}}_{.}^{ff.spp}$). **(B)** Bar plots showing functional annotation of the most HE genes, ${\text{DE}}_{.}^{ff.spp}$genes, and the 16 genes identified as unambiguous cases of non-parent of origin expression in the hybrid triticale mildew, based on conserved protein domain annotation, and subcellular localization prediction (see section Materials and Methods).

Our initial multidimensional scaling analysis indicated significant differences in gene expression between the three mildew *ff.spp*, suggesting there is a substantial number of genes with a *forma specialis*-specific expression level. Therefore, we studied differential gene expression between the three *ff.spp*., and we were particularly interested in comparing gene expression in the hybrid *B.g. triticale* to that of the wheat and rye mildew donors. We used the same criteria described earlier in this work for defining the thresholds for differential expression between wheat, rye, and triticale mildews. In total we found 292 DE genes (hereafter referred to as DE^*ff.spp*^) and most of these are found between *B.g. tritici* and *B.g. secalis* (257, hereafter called DE^wr^ genes, 146 putative effectors, 56.8%). We found less differences between triticale and wheat mildews (58 DE genes, 35 putative effectors, 60.3%) as compared to the difference between triticale and rye mildews (175 DE genes, 107 putative effectors, 61.1%) (Table 4, Figure 6). Analyses performed with the mapping obtained using different stringencies gave very similar results, with 85–94% of the genes identified as DE being the same irrespective of the mapping strategy (see Supplementary Table 8). In all cases the identified DE genes were highly enriched in putative effectors. We found 10 genes that are DE between the three *formae speciales* (Figure 7A), 144 genes that are DE between *B.g. secalis* and the two other *formae speciales* (Figures 7B,C), only five genes being DE between *B.g. triticale* and the two other *formae speciales* (Figure 7D) and 29 genes that are differentially expressed between *B.g. tritici* and the two other *formae speciales* (Figure 7E). Using the same GO and protein domain annotation approach described in previous sections, we found no statistically significant enrichment in any GO term or putative protein domains (see Supplementary Table 9, Supplementary Data Sheet 2). To further corroborate this enrichment of DE^*ff.spp*^ genes in candidate effectors, we applied different thresholds of log_2_FC to the same RNAseq pipeline (Log_2_FC > 1, Log_2_FC > 2 and Log_2_FC > 2.5, see section Materials and Methods) and we consistently found a significant enrichment in putative effectors irrespective of the chosen Log_2_FC threshold (Supplementary Table 10). We then compared the most HE genes and DE^*ff.spp*^ genes for gene content and predicted protein function. We found that most of the genes are unique to one of the sets (see Figure 8A) and that the two sets of genes encode for distinctly different protein functions. For the most HE genes we found that 34.7% encoded for proteins related to the secretome (i.e., putative effectors, non-effector secreted proteins, and proteins involved in secretion, see section Materials and Methods), while another 34.7% encoded for proteins related to ribosomal or mitochondrial functions (see Figure 8B, Supplementary Table 11). In contrast, DE^*ff.spp*^ genes mainly encoded for proteins related to the secretome (62.4%) including a large proportion of putative effectors (56.9%) and only 6.2% were related to the ribosome or the mitochondrion (see Figure 8B, Supplementary Table 11). This indicates that differences between *formae speciales* are mainly found in the secretome.

**Table 4:** Summary of the differentially expressed genes between wheat, rye, and triticale mildew *formae speciales*.

	**Differentially expressed**			
**Comparison**	**Genes^a^**	**Effectors^b^**	**Effectors (%)^c^**	***p*-value^d^**
*B.g. tritici* vs. *B.g. secalis* (DE^wr^)	257	146	56.8	<2.2e-16
*B.g. tritici* vs. *B.g. triticale*	58	35	60.3	<2.2e-16
*B.g. secalis* vs. *B.g. triticale*	175	107	61.1	<2.2e-16

a Total number of genes differentially expressed.

b Number of differentially expressed candidate effectors.

c Percentage of candidate effectors among differentially expressed genes.

d *P-value of the Fisher's exact test for enrichment in candidate effectors. A gene set is considered enriched in effectors if p-value < 0.01*.

Taken together, these results indicate that the expression in the hybrid *B.g. triticale* is more similar to that of *B.g. tritici* than to *B.g. secalis*, thus reflecting the hybrid genome composition, with higher proportions (>80%) being inherited from of the *B.g. tritici* donor (Menardo et al., 2016). In addition, most of the differences in gene expression between wheat, rye, and triticale mildews are contributed by genes encoding putative secreted proteins (mainly putative effectors) and proteins involved in the secretory pathway. This suggests that host adaptation in mildews can be based on transcriptional reprogramming of the secretome and selective activation of effector genes in a *forma specialis*-specific manner.

### Gene expression in the hybrid triticale mildew is conserved with the donor

As shown in the previous section, gene expression in the hybrid *B.g. triticale* is more similar to that of wheat mildew, consistent with the genomic composition of triticale mildew (Menardo et al., 2016). Therefore, we further assessed to what extent gene expression in the two hybrid isolates is correlated to the sequence origin of the gene which can be either inherited from the wheat or rye mildews. Thus, we based our analysis on the 257 genes which are differentially expressed between rye and wheat mildews (see Supplementary Figure 4) as follows: (i) we classified gene expression levels of the 257 genes in the hybrid into *B.g. tritici*-like or *B.g. secalis*-like, based on a the previously described thresholds for differential gene expression (see section Materials and Methods), (ii) we assessed the possible wheat or rye mildew origin of gene coding and promoter sequences in each of the two *B.g. triticale* isolates, based on sequence comparison and phylogenetic clustering (see section Materials and Methods), and (iii) we assessed to what extent gene expression levels in the hybrid can be correlated to the origin of the gene coding and/or promoter sequences.

In a first step, we were able to assign a *B.g. tritici*-like or *B.g. secalis*-like expression for 188 and 196 out of the 257 DE^wr^ genes in THUN-12 and T3-8, respectively. In THUN-12, 146 genes showed a *B.g. tritici*-like expression, 42 genes had similar expression to *B.g. secalis*, and 15 showed a hybrid specific level of expression. Similarly, in T3-8, 144 genes had a *B.g. tritici*-like expression, 52 exhibited a *B.g. secalis*-like expression, and 11 showed a hybrid-specific expression level (Table 5, Supplementary Figure 5). For 54 genes in THUN-12, and 50 in T3-8, the expression level was neither significantly different from *B.g. tritici*, nor *B.g. secalis* (see section Materials and Methods), therefore they could not be assigned to either of the *B.g. tritici*-like or *B.g. secalis*-like expression groups.

**Table 5:** Analysis of correlation between sequence origin and expression levels in the *B.g. triticale* hybrid of 257 genes differentially expressed between wheat and rye mildews.

	**Similar to *B.g. tritici*^a^**		**Similar to *B.g. secalis*^b^**		**Unique to *B.g. triticale*^c^**		**Undefined^d^**	
	**THUN-12**	**T3-8**	**THUN-12**	**T3-8**	**THUN-12**	**T3-8**	**THUN-12**	**T3-8**
Expression	146 (77.7%)	144 (73.5%)	42 (22.3%)	52 (26.5%)	15	11	54	50
Genomic sequence	133 (85.3%)	132 (84.1%)	23 (14.7%)	25 (15.9%)			101	100
Promoter sequence	131 (82.4%)	134 (85.5%)	28 (17.6%)	23 (14.6%)			98	100

a Number of genes among the 257 selected gene (DE^wr^) subset showing similarity to B.g. tritici based on expression level, origin of the gene sequence, or origin of the promoter sequence.

b *Number of genes among the 257 selected gene subset showing similarity to B.g. secalis based on the same criteria listed in ^a^*.

c Number of genes with an expression level unique to the hybrid B.g. triticale.

d *Number of genes with undefined origin of expression or sequence. All numbers are given for both B.g. triticale isolates THUN-12 and T3-8 as well as a percentage of the 257 considered genes between brackets*.

**Figure 9:**
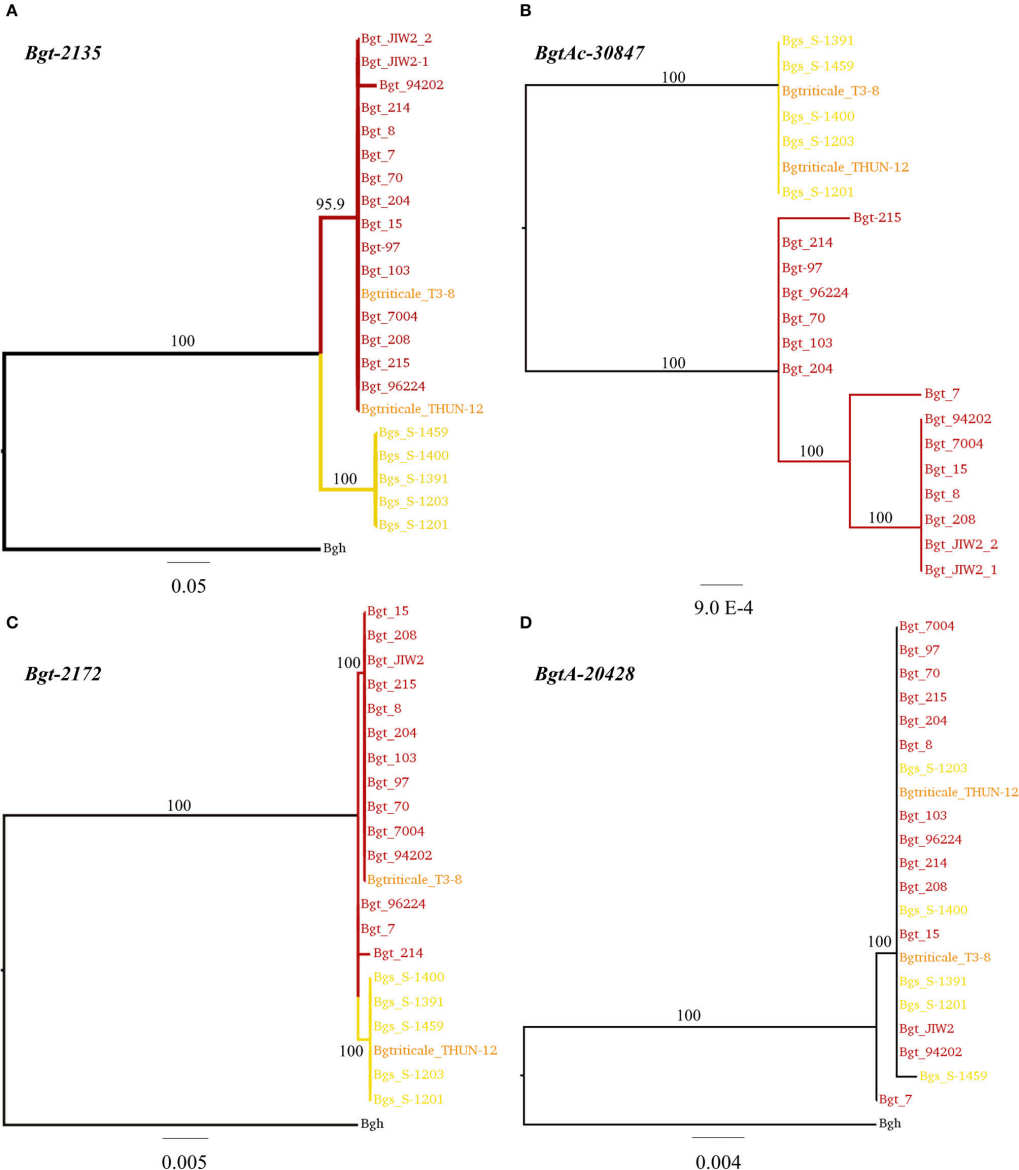
Phylogenetic analysis of four genes differentially expressed between wheat and rye mildew based on 23 *Blumeria graminis* genomes. Selected examples of *B.g. triticale* genes for which phylogeny data was used to assign the wheat or rye mildew origin of the gene. The trees were inferred with nucleotide sequences with 10,000 bootstraps. The scale bar indicates a measures of expected substitution per site. Bootstrap values are indicated on the branches. In **(A)** the origin of the gene *Bgt-2135* was assigned as *B.g. tritici* in both triticale isolates T3-8 and THUN-12. In **(B)** the origin of *Bgt-30847* was assigned as *B.g. secalis* in both isolates. In **(C)** the gene *Bgt-2172* was classified as originating from *B.g. tritici* in T3-8, and *B.g. secalis* in THUN-12. Finally, an example of a gene with undefined origin is depicted in **(D)** for *BgtA-20428*. In all cases, the sequence of the direct homolog from barley powdery mildew (Bgh) was used as an outgroup.

In a second step, we assessed the possible genomic origin of the 257 DE^wr^ genes in THUN-12 and T3-8. We used a phylogenetic approach to identify clusters of highly similar sequences (see section Materials and Methods for detailed procedure) and sequence information from 22 mildew isolates including: the two *B.g. triticale* isolates THUN-12 and T3-8, 14 of *B.g. tritici* (including 96224, 94202, and JIW2), 5 of *B.g. secalis* (including S-1391 and S-1459), and the reference barley powdery mildew genome as an outgroup (see Supplementary Table 2 and section Materials and Methods) (Spanu et al., 2010; Wicker et al., 2013; Menardo et al., 2016). We were able to assign a distinct wheat or rye mildew genomic origin for 156 genes in THUN-12, and 157 genes in T3-8, out of the 257 DE^wr^ genes. In THUN-12, 133 genes originated from *B.g. tritici* (Figure 9A) and 23 from *B.g. secalis* (see Figures 9B,C), and the results in T3-8 are very similar with 132 genes originating from *B.g. tritici* (Figures 9A,C) and 25 from *B.g. secalis* (Figure 9B). For 101 genes in THUN-12 and 100 in T3-8, it was not possible to distinguish which *forma specialis* they originate from based on our phylogenetic analysis (see Figure 9D, Table 5, Supplementary Figure 6, and section Materials and Methods for detailed procedure).

In a third step, we assessed the possible wheat or rye mildew origin of the promoter sequences of the 257 DE^wr^ genes in THUN-12 and T3-8. We defined the promoter sequence as the 1kb region upstream of the gene start, and used the same phylogenetic approach described above to classify those sequences based on their origin. We were able to assign a donor genome for 159 sequences from THUN-12 and 157 from T3-8. In THUN-12, 131 promoter sequences could be assigned to *B.g. tritici* and 28 to *B.g. secalis*. Similarly, in T3-8, 134 originated from the *B.g. tritici* donor genome and 23 from *B.g. secalis*. Finally, we could not determine a genome of origin for 98 gene promoters in THUN-12 and 100 in T3-8 (Table 5).

Based on the data obtained from the three approaches described above (i.e., expression level, origin of the coding sequence, and origin of the promoter sequence), we compared the composition of our subset of 257 DE^wr^ genes to that of the genomes of the studied triticale isolates. For THUN-12, we found that 15% of the genes, and 18% of the promoters of the DE^wr^ genes originated from *B.g. secalis*, consistent with the overall proportion of the rye mildew genome in this isolate (17%, Menardo et al., 2016). Similarly, we found that 16% of the genes and 15% of the promoters in T3-8 originated from *B.g. secalis*, which is also consistent with the overall proportion of the rye mildew genome in this isolate (13%, Menardo et al., 2016). However, on the gene expression level, we found that 22% of the DE^wr^ genes in THUN-12, and 26% in T3-8 were expressed as in rye mildew, suggesting a bias toward a *B.g. secalis*-like gene regulation in the hybrid triticale mildew. In particular, we found 17 unambiguous cases where the origin of the gene did not correlate with its expression, and only three of these genes (*Bgt_avrF2_20, Bgt-2135*, and *Bgt-2708*) were common to both triticale isolates (Table 6). Of these 17 cases, two genes for which the origin of the promoter sequences could not be assigned, had the coding sequence of *B.g. secalis* and an expression level of *B.g. tritici*. The 15 remaining genes all had the coding sequence of *B.g. tritici* and an expression level similar to *B.g. secalis*. Of these 15 genes with a *B.g. secalis*-like expression, we were able to resolve a possible origin of the promoter for 9 cases. Of these, 8 had a *B.g. tritici* promoter inconsistent with their *B.g. secalis*-like expression, and only one gene, *Bgt_avrF2_20*, had a *B.g. secalis* promoter consistent with its expression level (Table 6). These results suggest that the bias toward a *B.g. secalis*-like gene regulation in the hybrid is not due to an enrichment in genes inherited from the rye genome, but to wheat mildew inherited genes that are regulated like rye mildew genes. They also suggest that such non-parent of origin gene expression in the hybrid might be highly influenced by the genetic background since most of the identified instances in triticale mildew are isolate specific.

**Table 6:** Analysis of correlation between sequence origin and expression levels in 17 unambiguous cases of *de novo* gene regulation in the hygrid triticale mildew.

	**Gene name**	**Expression^a^**	**Up-/down- regulation^b^**	**Genomic sequence^c^**	**Promoter sequence^d^**	**Isolate^e^**	**Subcellular localization^f^**	**Effector^g^**	**Effector family/annotation^h^**
1	*Bgt_avrF2_20*	*B.g. secalis*	−	*B.g. tritici*	*B.g. secalis*	THUN-12, T3-8	Secretory pathway	^*^	*AvrPm3^*a*2/*f*2^*
2	*Bgt-2135*	*B.g. secalis*	−	*B.g. tritici*	*B.g. tritici*	THUN-12, T3-8	Secretory pathway	^*^	−
3	*Bgt-2708*	*B.g. secalis*	−	*B.g. tritici*	*B.g. tritici*	THUN-12, T3-8	Secretory pathway	^*^	−
4	*Bgt_BCG-6*	*B.g. secalis*	+	*B.g. tritici*	*B.g. tritici*	THUN-12	Secretory pathway	^*^	*SvrPm3^*a*1/*f*1^*
5	*Bgt-3010*	*B.g. secalis*	+	*B.g. tritici*	*B.g. tritici*	THUN-12	Sugar metabolism		Mannitol dehydrogenase C-terminal domain
6	*BgtE-20100*	*B.g. secalis*	−	*B.g. tritici*	*B.g. tritici*	THUN-12	Secretory pathway	^*^	13
7	*BgtAc-31565*	*B.g. secalis*	−	*B.g. tritici*	Undefined	THUN-12	NA	^*^	
8	*BgtASP-21288*	*B.g. secalis*	−	*B.g. tritici*	Undefined	THUN-12	Membrane	^*^	Proteolipid membrane potential modulator
9	*Bgt_BCG-5*	*B.g. secalis*	−	*B.g. tritici*	*B.g. tritici*	T3-8	Secretory pathway	^*^	*SvrPm3^*a*1/*f*1^*
10	*Bgt-3045*	*B.g. secalis*	−	*B.g. tritici*	*B.g. tritici*	T3-8	Membrane	^*^	MARVEL domain-containing proteins
11	*BgtE-20002*	*B.g. secalis*	−	*B.g. tritici*	*B.g. tritici*	T3-8	Secretory pathway	^*^	*AvrPm3^*a*2/*f*2^*
12	*Bgt_BCG-1*	*B.g. secalis*	−	*B.g. tritici*	Undefined	T3-8	Secretory pathway		*SvrPm3^*a*1/*f*1^*
13	*BgtAcSP-31482*	*B.g. secalis*	−	*B.g. tritici*	Undefined	T3-8	Secretory pathway	^*^	9
14	*BgtE-10129*	*B.g. secalis*	−	*B.g. tritici*	Undefined	T3-8	Secretory pathway		56
15	*BgtE-20051*	*B.g. secalis*	−	*B.g. tritici*	Undefined	T3-8	Secretory pathway		13
16	*Bgt-2172*	*B.g. tritici*	−	*B.g. secalis*	Undefined	THUN-12	NA		
17	*Bgt-20216*	*B.g. tritici*	+	*B.g. secalis*	Undefined	T3-8	NA		Pseudogene

a Classification of gene regulation based on B.g. tritici or B.g. secalis-like expression levels (see text and section Materials and Methods).

b Classification of gene expression levels based on upregulation (+) or downregulation (−) in the hybrid B.g. triticale as compared to B.g. tritici (genes 1–15) and to B.g. secalis (genes 16 and 17).

c Origin of the genomic sequence.

d Origin of the promoter sequence which was defined as the 1kb region upstream gene start (see section Materials and Methods).

e Triticale isolates where each case of de novo regulation was found.

f Prediction of subcellular localization using TargetP (REF target, see section Materials and Methods).

g Genes encoding for candidate secreted effectors.

h *Functional annotation based on effector gene families, or putative protein function of non-effector genes. For readability, if the expression, genomic sequence or promoter sequence of one gene is originating from B.g. tritici or from B.g. secalis, the corresponding cell has been highlighted in red or yellow respectively*.

We found that 11 out of the 17 unambiguous cases where the origin of the genes did not correlate with expression encode candidate effectors. Interestingly, all 11 putative effectors encode wheat mildew genes that are expressed like rye genes, and all except one (*Bgt_BCG-6*) are downregulated in the hybrid triticale as compared to their expression in the wheat mildew donor genome (Table 6). Of the 11 genes, two are members of the effector family encoding the *B.g. tritici* avirulence gene *AvrPm3*^*a*2/*f*2^ (including *Bgt_avrF2_20*), one is the direct homolog of the wheat mildew *R* gene suppressor *SvrPm3*^*a*1/*f*1^, and two additional ones (*Bgt_BCG-5* and *Bgt_BCG-6*) are members of the *SvrPm3*^*a*1/*f*1^ effector family (Table 6). These results substantiate the hypothesis that mildew effectors, in particular those encoded within *Avr* and *Svr* gene families, likely play an important role in host-adaptation. Furthermore, we postulate that these putative effectors are possible targets of immune receptors encoded by the rye genome or the rye sub-genome in the hybrid triticale host, thus explaining why these putative effectors are expressed to lower levels in *B.g. secalis* and *B.g. triticale* as compared to *B.g. tritici* during compatible interactions with their respective adapted hosts.

To summarize, our results indicate that for most of the genes in the mildew hybrid, there is high level of conservation of gene regulation based on the *B.g. tritici* or *B.g. secalis* origin of the gene and the promoter sequences. However, we found a higher contribution of *B.g. secalis* to gene regulation in the hybrid as compared to the overall proportions of the rye mildew genome in *B.g. triticale*. We have also found a few but prominent examples of non-parent of origin expression of putative effector genes encoded within gene families commonly involved in cultivar adaptation in wheat mildew.

## Discussion

### The combination of transcriptomics and genetic diversity data allows inter-*Formae speciales* comparisons

In this study, we compared seven isolates from three *formae speciales*, namely wheat, rye, and triticale powdery mildews. The three *ff.spp*. cannot be distinguished based on morphological or cytological criteria. At the genomic level, *B.g. secalis* is relatively distant from *B.g. tritici*, while the *B.g. triticale* genome shows a hybrid architecture with segments originating from the wheat mildew donor and others from the rye mildew donor, with a very variable origin of the different segments in different isolates (Menardo et al., 2016). Many published RNAseq studies compared gene expression levels of the same fungal genotype under different conditions, at different times after infection, or between compatible and incompatible interactions (Cantu et al., 2013; Hacquard et al., 2013; Kellner et al., 2014; Palma-Guerrero et al., 2016; Sonah et al., 2016). In our work, we used a reference genome-based approach allowing both intra- and inter- *forma specialis* comparisons. Such comparisons of gene expression in different genotypes are challenging because the analysis is based on a reference genome while taking into account the variation in gene content between genotypes. In our case, the genomes of the wheat, rye, and triticale mildews are similar enough to allow the mapping of genomic and RNA sequencing reads on the *B.g. tritici* reference. However, high levels of sequence polymorphism between *formae speciales*, including gene duplications and losses must be accounted for. To do so, we developed a series of analyses based on the available genetic diversity of the mildew genomes. First, we established a method for identifying genes affected by presence/absence polymorphisms based on genome sequence coverage that allowed us to identify genes from our reference genomes that are absent in other *ff.spp*. Second, the considerable sequence diversity allowed us to establish a phylogeny-based method to determine the gene content in the triticale mildew based on the wheat or rye mildew origin of the sequences. Thus, our work demonstrates ways of using the genetic diversity of mildew isolates to resolve the genomic complexity of the mildew genomes for robust comparative transcriptome studies.

Our results suggest that many genes and putative effectors specific to *B.g. secalis* are missing from the *B.g. tritici* reference genome and that the number of putative effectors in *B.g. secalis* is underestimated in our analyses. This would explain why we have systematically observed lower levels of enrichment in putative effector genes in both HE and DE gene sets from *B.g. secalis* as compared to the other *ff.spp*. (Figure 3, Tables 2, 3). It is possible that a substantial number of putative effectors are yet to be annotated in the rye mildew genome, which will require the production of a *B.g. secalis* reference genome assembly. We propose that this can be now achieved using long read sequencing technologies such as PacBio Sequencing (Rhoads and Au, 2015). In addition, we also found that the most highly expressed putative effectors of rye mildew had overall lower expression levels than the ones from wheat or triticale mildews (95% quantile, Table 1). It is very likely that *B.g. secalis*-specific and highly expressed putative effectors have no direct homolog in the reference genome and are absent from our analysis. Such subspecies-specific gene and sequence variation is reminiscent of previous studies in *Magnaporthae oryzae* (Liao et al., 2016), rust fungi (Duplessis et al., 2011), and barley powdery mildew (Hacquard et al., 2013) who reported similar differences in the effector repertoire among different pathogen isolates, subspecies, or lineages, suggesting a role in cultivar and host adaptation.

### Identification of a core-set of highly expressed candidate effector genes in wheat, rye, and triticale mildews

In this study we identified a core-set of genes that are highly expressed in wheat, rye, and triticale mildews suggesting that there is a common mechanism of host infection among mildew *formae speciales*. This hypothesis is further substantiated by the fact that the highly expressed genes are enriched in candidate effectors. It has been proposed that candidate effector genes expressed at early stage of infection are involved in suppressing host immunity, in particular PAMP-triggered immunity. In *Pseudomonas syringae*, a bacterial pathogen that can infect a wide range of plant species, the majority of the encoded type III effectors have been shown to suppress plant immunity (Shan et al., 2008; Xiang et al., 2008; Guo et al., 2009). In barley powdery mildew, the putative effector BEC1054 was shown to be important for mildew virulence based on a reduction of 60% of the ability of the mildew to form haustoria after HIGS downregulation (Pliego et al., 2013). Also, BEC1054 was shown to target several barley proteins including a pathogenesis-related-5 protein isoform further substantiating its role in suppressing host immunity (Pennington et al., 2016b). Interestingly, we found 7 members of the BEC1054 family in the most HE genes, suggesting that they play an important role in virulence across several mildew *formae speciales*. Another prominent example of highly expressed effectors is *SvrPm3*^*a*1/*f*1^, the mildew encoded suppressor of the wheat *Pm3* resistance gene (Bourras et al., 2015; Parlange et al., 2015), and the two known *B.g. tritici* avirulence genes *AvrPm2* and *AvrPm3*^*a*2/*f*2^ (Bourras et al., 2015; Praz et al., 2017).

Thus, our results identified a set of highly expressed candidate effector genes from three mildew *formae speciales*, including prominent examples of *bona fide* effectors, plant immunity suppressors, and *R* gene avirulence targets. We propose that candidate effector genes that are highly expressed in different *formae speciales* might share redundant functions in virulence based on targeting highly conserved proteins in the wheat, rye, and triticale host. These putative effectors might encode important virulence factors and yet uncharacterized AVR proteins, and we speculate that identification of their plant targets might provide new routes for achieving resistance.

### Quantitative differences in gene expression suggest possible role of candidate effector genes in host adaptation

We found that the genes differentially expressed between isolates of the same *forma specialis* are enriched in candidate effectors. One prominent case in *B.g. tritici* is *SvrPm3*^*a*1/*f*1^ (*Bgt_BCG-1*), a gene involved in suppressing the wheat *Pm3* race-specific resistance. It was shown that such suppression was based on a combination of the specific *SvrPm3*^*a*1/*f*1^ allele and a high expression level (Bourras et al., 2015). In *Leptosphaeria maculans*, RNAi silencing of the avirulence gene *AvrLm6* resulted in gain of virulence of the pathogen toward oilseed rape cultivars encoding its cognate *R* gene *Rlm6* (Fudal et al., 2007). Here, *SvrPm3*^*a*1/*f*1^ and *AvrLm6* provide two relevant examples of how differences in effector gene expression can contribute to cultivar adaptation of plant pathogenic fungi. Therefore, we propose that the identified DE genes between isolates of wheat, rye, or triticale mildews, are likely involved in cultivar adaptation, and probably encode for AVRs, SVRs, and important factors for pathogen fitness. Here, quantitative differences in effector gene expression represent an additional level of polymorphism allowing the pathogen to keep important virulence factors that can be transcriptionally activated to suppress resistance and increase pathogen fitness (e.g., SVRs, *bona fide* effectors), or inactivated to escape host recognition (e.g., AVRs).

Similarly, we found that the genes differentially expressed between *formae speciales* are also enriched in putative effectors suggesting they are involved in host adaptation in cereal mildews. In *Magnaporthe oryzae*, the role of effectors in host adaptation has been demonstrated based on significant differences in the effector complement between isolates specialized on japonica rice compared to those specialized on indica rice (Liao et al., 2016). In particular, indica adapted races of *M. oryzae* lacked several effectors including many encoding rice blast AVRs, with evidence suggesting that *AVR-Pia* in particular is acting as a host-specificity determinant in *M. oryzae* subspecies (Liao et al., 2016). Here, we propose that different levels of effector gene expression in different mildew *formae speciales* form an additional layer of regulation of host-specificity. Selective effector activation/inactivation at the transcriptional level in mildews will result in presence/absence variation of the effector at the protein level. Therefore, DE effectors between *ff.spp*. can possibly act as host specificity determinants in a way that is similar to the *M. oryzae* subspecies. Many of the DE^*ff.spp*.^ putative effectors encode for members of known mildew *Avr* and *Svr* families, including *AvrPm3*^*a*2/*f*2^, *AvrPm2, Avr*_*a*13_, and *SvrPm3*^*a*1/*f*1^. Therefore, we propose that host-adaptation in mildews can also be based on major resistance genes in the wheat, rye, and the triticale hosts. Indeed, previous work by Tosa using genetic crosses between mildew *formae speciales* showed mendelian segregation for host adaptation in the pathogen suggesting a genetic control of host specificity similar to that of *Avr-R* interactions (Tosa, 1989, 1992, 1994). It was also hypothesized that variation in pathogen effector repertoires, in particular those recognized by NLRs, is a driver of host adaptation in the pathogen (Schulze-Lefert and Panstruga, 2011). Here, selective activation of *Avr* and *Svr* genes instead of presence/absence variation at the gene level allows mildew *formae speciales* to escape host recognition while keeping important factors for virulence on their adapted host. This hypothesis is substantiated by the fact that R genes from different cereal species can effectively recognize *Avrs* across mildew *formae speciales*. Indeed, it was demonstrated that the wheat *Pm2* resistance gene recognizes the *AvrPm2* effector from *B.g. tritici*, as well as its direct homolog from the non-adapted rye mildew *forma specialis* (*BgsE-5845*) (Praz et al., 2017).

We propose that cultivar and host adaptation in mildews can be based on effector gene regulation. We also propose that selective activation of the effector repertoire is acting in a way reminiscent of presence/absence gene polymorphism, thus effectors can be either activated to suppress immunity or inactivated to escape recognition. Finally, consistent with the previous genetic evidence reported by Tosa (1989, 1992, 1994), our results provide additional evidence at the gene transcriptional level that host-adaptation in mildews is possibly based on major *R* genes in the host and the *Avr/Svr* composition of the pathogen.

### Suppression of non-host recognition by effector gene downregulation as a possible mechanism for host adaptation in triticale mildew

The hybridization event between wheat and rye mildew resulted in a hybrid *forma specialis B.g. triticale* with a mosaic genome structure made of segments from the wheat and rye donors (Menardo et al., 2016). The overall proportions of the wheat and rye inherited sequences is rather consistent across triticale isolates with >80% corresponding to *B.g. tritici* segments and <20% to *B.g. secalis*. However, the specific sequences that are inherited from the donor genomes are highly variable with for instance only 66 out of the 7,138 annotated mildew genes that were consistently inherited from *B.g. secalis* in the analyzed isolates (Menardo et al., 2016). In our study we found that the proportions of genes and promoter sequences originating from *B.g. secalis* in our subset of 257 DE^wr^ genes are reminiscent of the genomic composition of the *B.g. triticale* isolates. In this subset, 23 and 25 genes were inherited from rye mildew in T3-8 and THUN-12, respectively, and only 10 genes were common to both isolates. Therefore, one hypothesis is that host adaptation in triticale mildew relies on a core set of genes with unique functions in host adaptation which can only be inherited from the wheat or rye mildew donors, plus another set of genes with redundant/conserved functions which can be inherited from both donor genomes irrespective of the *formae specialis*. We also found that at the transcriptional level the proportions of genes expressed like rye-mildew genes was higher than the proportions of the rye mildew genome segments in the triticale mildew isolates, thus indicating a bias toward a *B.g. secalis*-like expression in the hybrid. These results indicate that in addition to the segmental genome structure in the hybrid, there is transcriptional reprogramming revealing a contribution higher than expected of the rye mildew genome to host-adaptation of *B.g. triticale*. We propose that this additional contribution is based on transcriptional reprogramming of genes that are inherited from wheat mildew and regulated as rye mildew genes in the hybrid.

In the 257 DE^wr^ genes we closely analyzed, we identified 17 unambiguous cases of transcriptional reprogramming where the *B.g. tritici* or *B.g. secalis* origin of the sequence did not correlate with the expression level *in B.g. triticale*. Of these, 15 were wheat mildew genes regulated like rye mildew-genes in the hybrid triticale, and only two showed the opposite pattern. It is striking that all 11 cases of putative effector genes showing non-parent of origin expression levels corresponded to wheat mildew genes that are expressed like rye mildew genes, and that 5 of these belong to effector gene families containing known mildew determinants of cultivar adaptation. In all cases except for one gene (*Bgt_BCG6*), non-parent of origin regulation in the hybrid corresponded to a downregulation of a wheat mildew inherited putative effector gene to significantly lower levels similar to those of its direct homolog in rye mildew. All together these results suggest that host-adaptation in triticale mildew is based on downregulation of effectors inherited from wheat mildew, possibly resulting in the evasion of host recognition. Since these effectors are upregulated in wheat mildew during compatible interaction with its adapted host, it is unlikely that they are recognized by the wheat immune receptors/factors responsible for the recognition of non-adapted mildews. Therefore, we postulate that these effectors are likely acting as determinants of non-host resistance of rye and triticale immunity to wheat powdery mildew.

To conclude, we propose that the molecular basis of host-adaptation of triticale mildew is mainly based on differences in the secretome, and in particular on differential effector gene expression. We hypothesize that some effectors inherited from *B.g. tritici* in the hybrid *B.g. triticale* can be recognized by immune receptors/factors encoded in the rye sub-genome of the triticale host. Therefore, adapted triticale mildew hybrids have evolved a mechanism for host-adaptation based on specific down-regulation of those effectors involved in non-host resistance of rye to wheat powdery mildew. Finally, we also propose that *Avr* and *Svr* effector gene families in the pathogen and major *R* genes in the host, play and important role in host adaptation which can be controlled by *Avr-R-Svr* interactions.

## Accession numbers

The genome reference, the corresponding annotation (GFF), and all gene sequences (CDS) used in this study can be accessed at GenBank under the accession number PRJEB23548. All transcriptomic sequences used for this study can be accessed at the Gene Expression Omnibus (GEO) under accessions GSE73399 and GSE108405.

## Author contributions

CP, SB, and BK designed the study. CP, FM, MM, and SB performed experiments. CP, FM, MR, and TW provided analysis tools. CP and FM performed analyses. CP, SB, and BK wrote and edited the manuscript.

### Conflict of interest statement

The authors declare that the research was conducted in the absence of any commercial or financial relationships that could be construed as a potential conflict of interest.
