# Supplementary material for: Non-parent of Origin Expression of Numerous Effector Genes Indicates a Role of Gene Regulation in Host Adaption of the Hybrid Triticale Powdery Mildew Pathogen
Source: Front Plant Sci. 2018 Jan 30;9:49. doi: 10.3389/fpls.2018.00049 (PMC5797619; doi:10.3389/fpls.2018.00049)
Supplement: Supplementary file 1 [file SupplementaryMaterial.PDF]

Supplementary Material

Non parent-of-origin expression of numerous effector genes indicates a role of gene regulation in host adaption of the hybrid triticale powdery mildew pathogen

Coraline R. Praz[1], Fabrizio Menardo[1], Mark D. Robinson[2], Marion C. Müller[1], Thomas Wicker[1], Salim Bourras[1]* and Beat Keller[1]*

*** Correspondence:**

Beat Keller
bkeller@botinst.uzh.ch

Salim Bourras
s.bourras@botinst.uzh.ch

1 Supplementary Data

Supplementary Data 1. R Script for the different gene expression analyses.

Supplementary Data 1 is available as an additional file.

Supplementary Data 2. Gene Ontology Annotation of the *Blumeria graminis* genes.

Supplementary Data 2 is available as an additional file.

Supplementary Data 3. Sequences of the 595 candidate effector genes defined in Praz et al. (2017) and used in this study.

Supplementary Data 3 is available as an additional file.

Supplementary Data 4. Protein sequences of genes of known effector families (see S3 Table).

Supplementary Data 4 is available as an additional file.

2 Supplementary Figures and Tables

2.1 Supplementary Figures

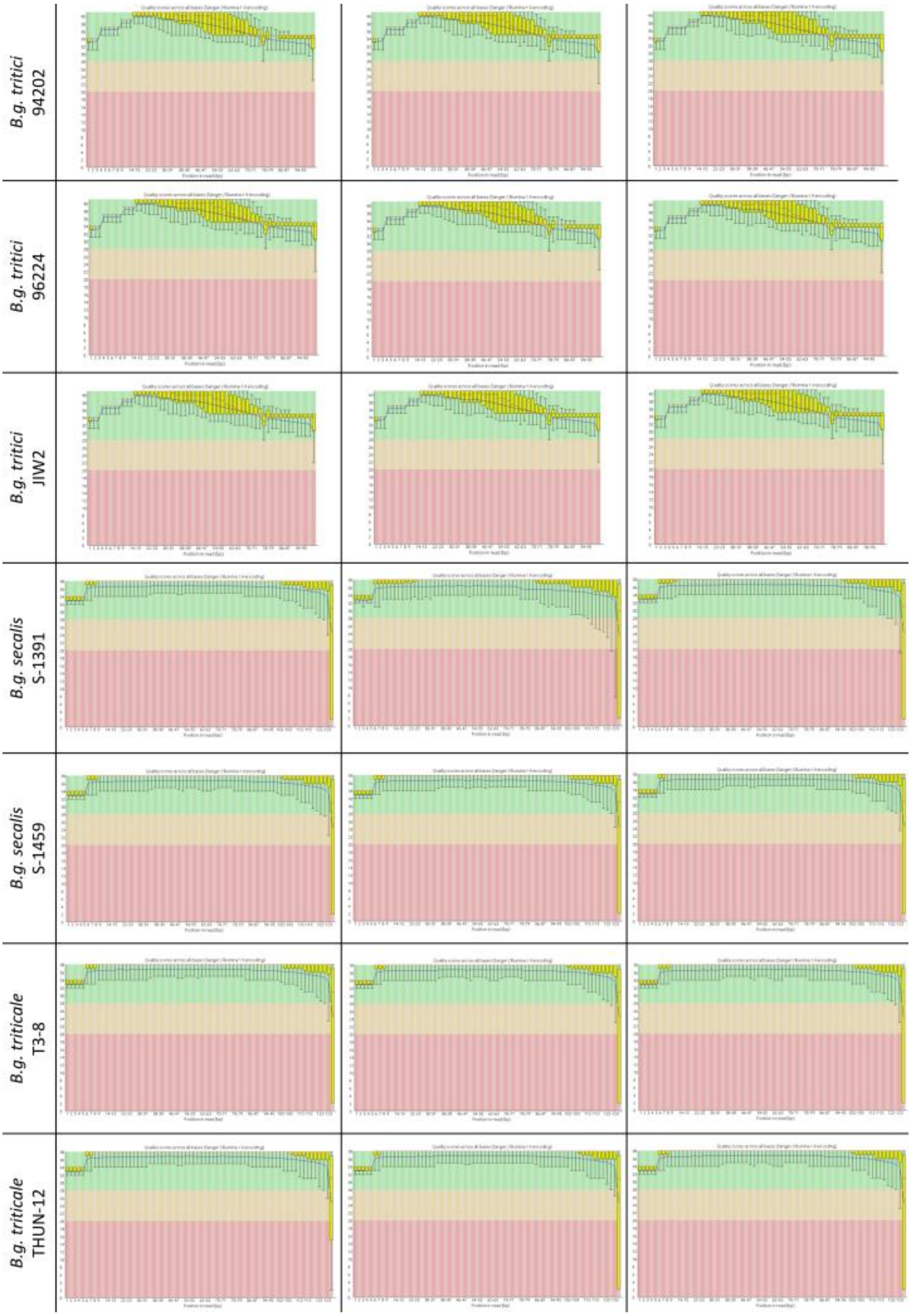

Supplementary Figure 1: Quality control of the RNA-seq libraries.

The per base sequence quality of the 21 RNA-seq libraries obtained with FastQC are depicted. Each row is one isolate and the columns are the replicates.

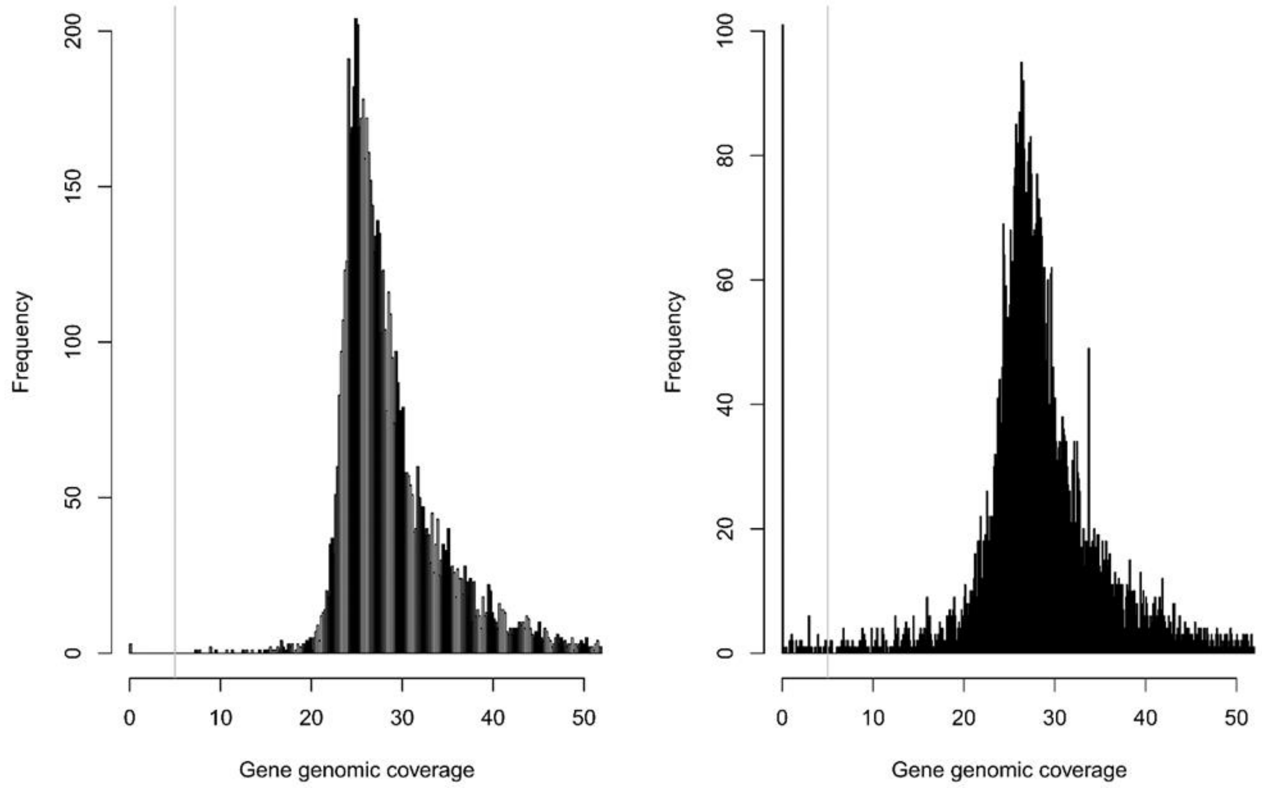

Supplementary Figure 2. Coverage distribution of individual genes in the isolates *B.g. tritici* 96224 and *B.g. secalis* S-1391.

(A) Coverage distribution of individual genes in the *B.g. tritici* isolate 96224. **(B)** Coverage distribution of individual genes in the *B.g. secalis* isolate S-1391. The coverage is indicated in reads per kilo base pairs per million reads on the ‘x’ axis. The number of genes is indicated on the ‘y’ axis. The threshold of 5x coverage is indicated by a vertical grey line. All genes with a coverage lower than the 5x threshold were considered as absent in the corresponding isolate (see Methods).

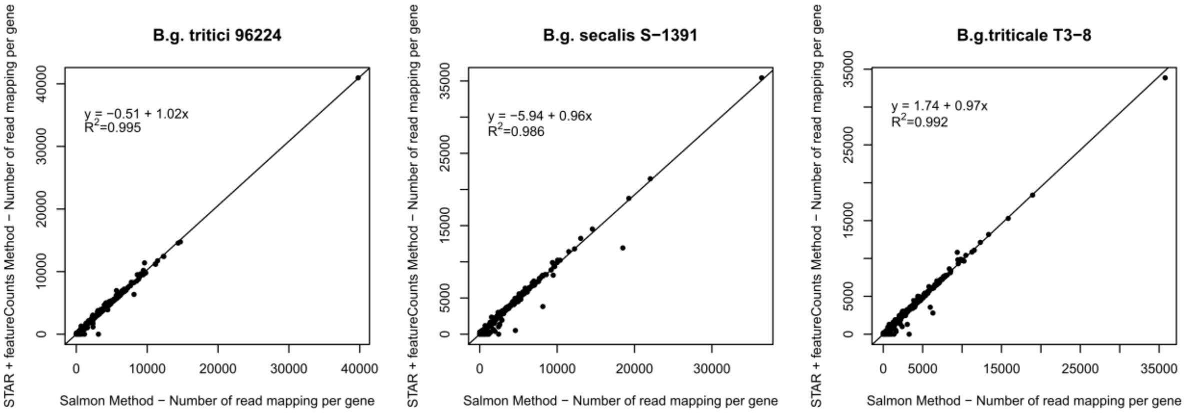

Supplementary Figure 3: Correlation between expression values obtained with the STAR + featureCounts and the Salmon based methods.

Read counts of 6,203 genes are plotted as a function of expression estimate values obtained with Salmon on the x-axis, and the count values obtained with STAR and featureCounts on the y-axis. One isolate from each mildew *forma specialis* is depicted, and the corresponding plot is labelled accordingly.

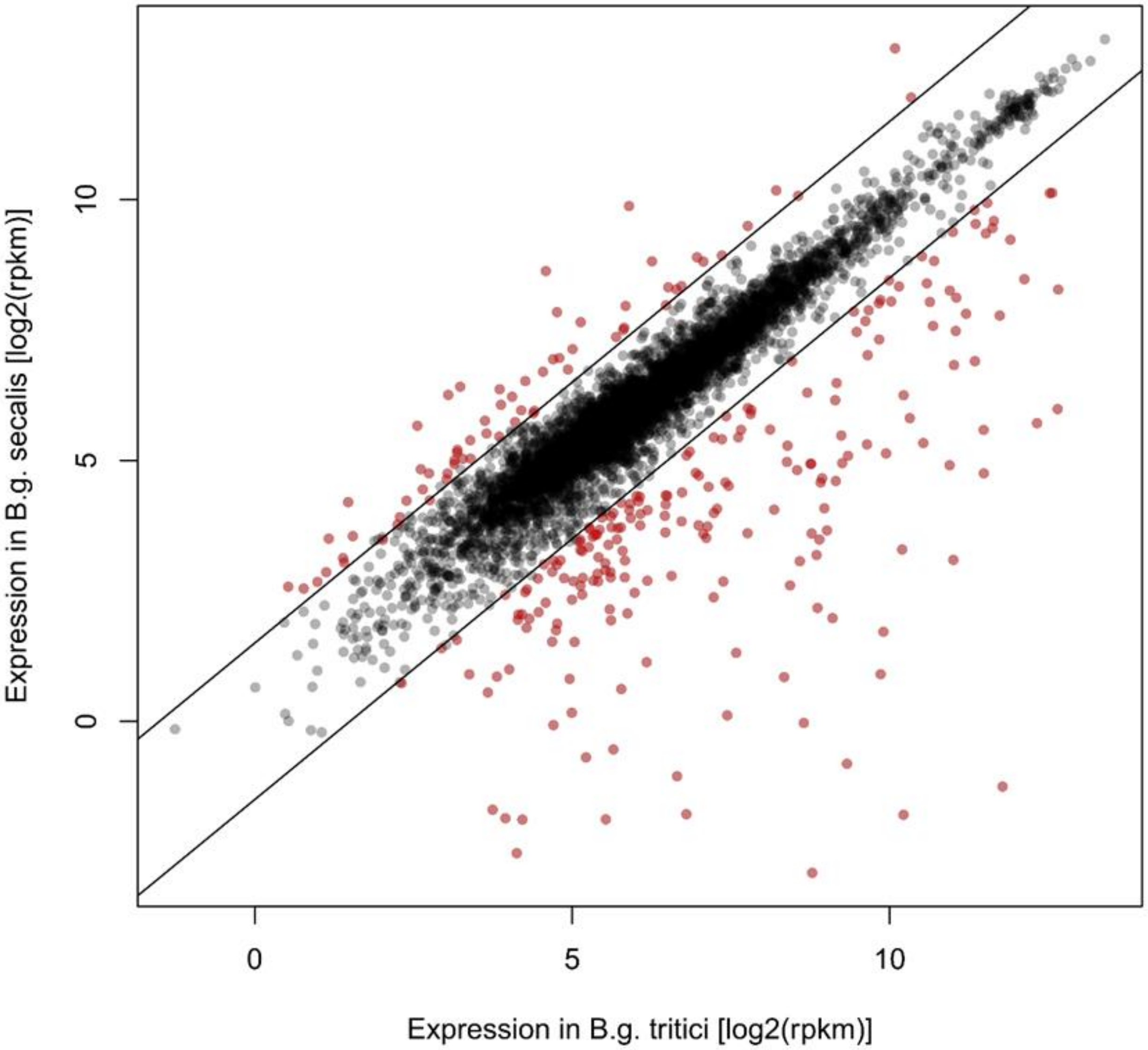

Supplementary Figure 4. Genome-wide comparison of gene expression in *B.g. tritici* and *B.g. secalis.*

The expression values of 6,203 genes are plotted as the mean expression in the *B.g.tritici* isolates on the x-axis, and the mean expression in the *B.g. secalis* isolates on the y-axis. The two black lines represent the threshold for differential expression of log2FC = ±1.5. Genes not passing this threshold are depicted as black dots inside the interval delimited by the |1.5| threshold lines. The 257 genes DE between *B.g. tritici* and *B.g. secalis* (log2FC > |1.5|) are depicted as red dots outside the interval delimited by the |1.5| threshold lines.

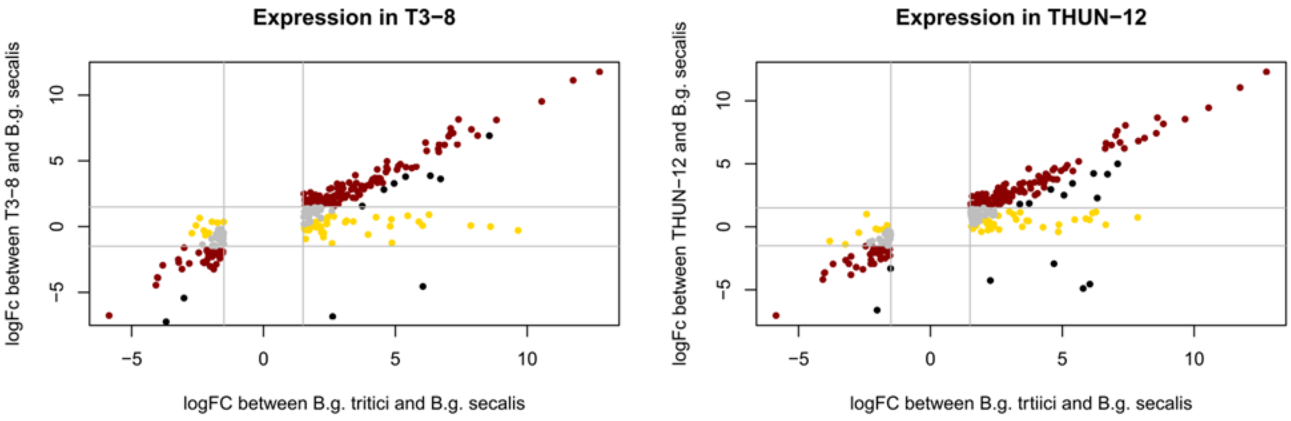

Supplementary Figure 5. Expression of the 257 genes differentially expressed between *B.g. tritici* and *B.g. secalis* in the *B.g. triticale* isolates T3-8 and THUN-12.

Each dot represents one of the 257 genes DE between *B.g. tritici* and *B.g. secalis*. The x-axis indicates logFC expression difference between *B.g. tritici* and *B.g. secalis*. The y-axis indicates logFC expression difference between *B.g. secalis* and the *B.g. triticale* isolate T3-8 (left panel) or the *B.g. triticale* isolate THUN-12 (right panel). Red dots represent genes with a *B.g. tritici*-like expression in each isolate of *B.g. triticale*. Yellow dots represent genes with a *B.g. secalis*-like expression. Grey dots represent genes for with expressions levels that cannot be differentiated from those in *B.g. tritici* or *B.g. secalis*. Black dots represent genes that have a distinctly different expression level in the *B.g. triticale* isolate compared to the *B.g. tritici* and the *B.g. secalis* donors.

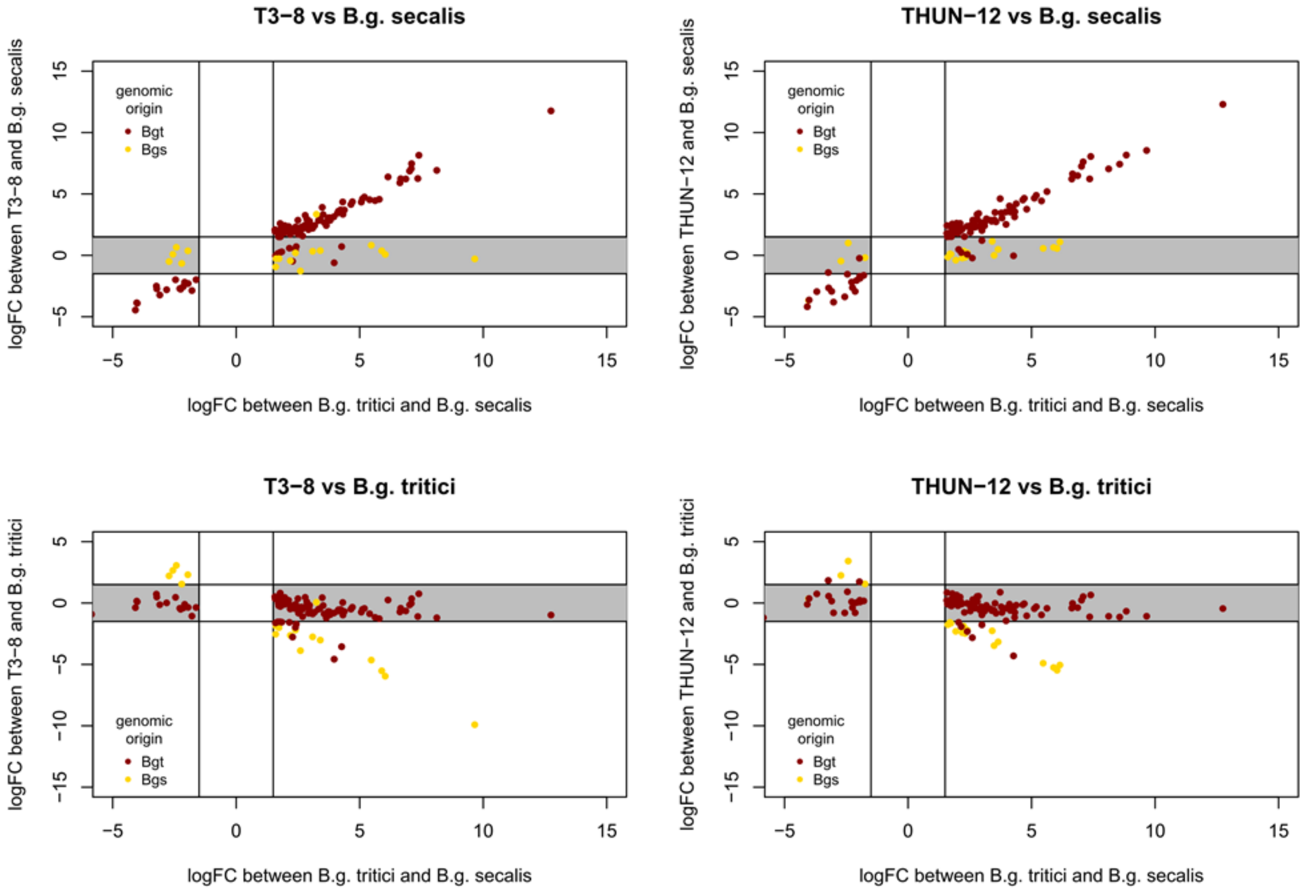

Supplementary Figure 6. Genomic origin and expression level of the genes that are differentially expressed between *B.g. tritici* and *B.g. secalis* (DE^{wr}) in the *B.g. triticale* isolates.

The x-axis indicates logFC expression difference between *B.g. tritici* and *B.g. secalis*. The y-axis indicates logFC expression difference between one *B.g. triticale* isolate (T3-8 on the left and THUN-12 on the right respectively) and either *B.g. secalis* (upper row) or *B.g. tritici* (lower row). Red dots represent genes that originate from *B.g. tritici* in the respective *B.g. triticale* isolate. Yellow dots represent genes originating from *B.g. secalis*. Genes for which the origins could not be defined are not represented. In the upper row, red dots inside the grey area represent genes with a *B.g. secalis*-like expression but a *B.g. tritici* origin of the sequence. In the lower row, yellow dots inside the grey area represent genes with a *B.g. tritici*-like expression but originating from *B.g. secalis*.

2.2 Supplementary Tables

Supplementary Table 1. Summary of the RNA-Seq samples and libraries used in this study.

Sample	*Forma specialis*	Isolate	Biological replicate	Host	Host cultivar	Number of raw reads	Uniquely mapped reads	Uniquely mapped reads %	Reference	Accession number
94202_1	*B.g. tritici*	94202	1	Bread wheat	Chinese Spring	39047283	1866534	4,78	This study	GSE108405
94202_2	*B.g. tritici*	94202	2	Bread wheat	Chinese Spring	43576349	2810053	6,45	This study	GSE108405
94202_3	*B.g. tritici*	94202	3	Bread wheat	Chinese Spring	46286859	2087192	4,51	This study	GSE108405
96224_1	*B.g. tritici*	96224	1	Bread wheat	Chinese Spring	46039585	4043986	8,78	This study	GSE108405
96224_2	*B.g. tritici*	96224	2	Bread wheat	Chinese Spring	32536396	1897091	5,83	This study	GSE108405
96224_3	*B.g. tritici*	96224	3	Bread wheat	Chinese Spring	50446400	4097757	8,12	This study	GSE108405
JIW2_1	*B.g. tritici*	JIW2	1	Bread wheat	Chinese Spring	36342372	2168243	5,97	This study	GSE108405
JIW2_2	*B.g. tritici*	JIW2	2	Bread wheat	Chinese Spring	66964624	3821544	5,71	This study	GSE108405
JIW2_3	*B.g. tritici*	JIW2	3	Bread wheat	Chinese Spring	47837019	2568832	5,37	This study	GSE108405
S-1391_1	*B.g. secalis*	S-1391	1	Rye	Matador	79269850	3963296	5,00	This study	GSE108405
S-1391_2	*B.g. secalis*	S-1391	2	Rye	Matador	63229778	3333053	5,27	This study	GSE108405
S-1391_3	*B.g. secalis*	S-1391	3	Rye	Matador	90616597	3397398	3,75	This study	GSE108405
S-1459_1	*B.g. secalis*	S-1459	1	Rye	Matador	70457559	4008017	5,69	This study	GSE108405
S-1459_2	*B.g. secalis*	S-1459	2	Rye	Matador	78791318	4610685	5,85	This study	GSE108405
S-1459_3	*B.g. secalis*	S-1459	3	Rye	Matador	90786643	4832340	5,32	This study	GSE108405
T3-8_1	*B.g. triticale*	T3-8	1	Triticale	Timbo	75337592	4356982	5,78	Menardo et al, 2016	GSE73399
T3-8_2	*B.g. triticale*	T3-8	2	Triticale	Timbo	88421931	3267642	3,70	Menardo et al. 2016	GSE73399
T3-8_3	*B.g. triticale*	T3-8	3	Triticale	Timbo	71438448	3407449	4,77	Menardo et al. 2016	GSE73399
THUN-12_1	*B.g. triticale*	THUN-12	1	Triticale	Timbo	84489605	5700324	6,75	Menardo et al. 2016	GSE73399
THUN-12_2	*B.g. triticale*	THUN-12	2	Triticale	Timbo	70899954	3552159	5,01	Menardo et al. 2016	GSE73399
THUN-12_3	*B.g. triticale*	THUN-12	3	Triticale	Timbo	85299660	4602829	5,40	Menardo et al. 2016	GSE73399

Supplementary Table 2. Summary of the wheat, rye, triticale, and barley powdery mildew isolates and genomes used in phylogenetic analyses.

Isolate	*Forma specialis*	Genome Reference	Accession number
94202	*B.g. tritici*	Menardo et al. 2016	SRP062198
JIW2	*B.g. tritici*	Wicker et al. 2013	ASJL00000000.1
214	*B.g. tritici*	Menardo et al. 2016	SRP062198
8	*B.g. tritici*	Menardo et al. 2016	SRP062198
7	*B.g. tritici*	Menardo et al. 2016	SRP062198
70	*B.g. tritici*	Menardo et al. 2016	SRP062198
204	*B.g. tritici*	Menardo et al. 2016	SRP062198
15	*B.g. tritici*	Menardo et al. 2016	SRP062198
97	*B.g. tritici*	Menardo et al. 2016	SRP062198
103	*B.g. tritici*	Menardo et al. 2016	SRP062198
7004	*B.g. tritici*	Menardo et al. 2016	SRP062198
208	*B.g. tritici*	Menardo et al. 2016	SRP062198
215	*B.g. tritici*	Menardo et al. 2016	SRP062198
S-1400	*B.g. secalis*	Menardo et al. 2016	SRP062198
S-1391	*B.g. secalis*	Menardo et al. 2016	SRP062198
S-1459	*B.g. secalis*	Menardo et al. 2016	SRP062198
S-1203	*B.g. secalis*	Menardo et al. 2016	SRP062198
S-1201	*B.g. secalis*	Menardo et al. 2016	SRP062198
T3-8	*B.g. triticale*	Menardo et al. 2016	SRP062198
THUN-12	*B.g. triticale*	Menardo et al. 2016	SRP062198
DH-14	*B.g. hordei*	Spanu et al. 2010	GCA_000151065.1

Supplementary Table 3. Comparison of the mapping statistics for the three *formae speciales* obtained with four different mapping strategies.

Mapping[1]	Number of mismatches[2]	Number of multimappers[3]	Forma specialis[4]	% of uniquely mapped reads[5]	% of reads mapped to multiple loci[6]	% of reads mapped to too many loci[7]	% of all mapped reads[8]	% of reads assigned to a feature[9]
1	0.01	1	*B.g. tritici*	6.17%	0.00%	0.12%	6.17%	4.05%
1	0.01	1	*B.g. secalis*	5.15%	0.00%	0.07%	5.15%	3.58%
1	0.01	1	*B.g. triticale*	5.24%	0.00%	0.07%	5.23%	3.60%
2	0.04	1	*B.g. tritici*	6.23%	0.00%	0.14%	6.23%	4.07%
2	0.04	1	*B.g. secalis*	5.33%	0.00%	0.10%	5.33%	3.66%
2	0.04	1	*B.g. triticale*	5.33%	0.00%	0.09%	5.33%	3.64%
3	0.01	20	*B.g. tritici*	6.05%	0.09%	0.00%	6.15%	4.01%
3	0.01	20	*B.g. secalis*	4.79%	0.06%	0.00%	4.84%	3.39%
3	0.01	20	*B.g. triticale*	5.05%	0.05%	0.00%	5.10%	3.51%
4	0.04	20	*B.g. tritici*	6.23%	0.14%	0.00%	6.36%	4.08%
4	0.04	20	*B.g. secalis*	5.33%	0.10%	0.00%	5.43%	3.68%
4	0.04	20	*B.g. triticale*	5.33%	0.09%	0.00%	5.41%	3.65%

[1] Mapping strategy

[2] Number of mismatches allowed per read

[3] Number of multimappers allowed

[4] *Forma specialis*

[5] Percentage of reads mapping uniquely on the genome.

[6] Percentage of reads mapping to multiple loci on the genome that are considered in downstream analyses.

[7] Percentage of reads mapping to too many loci on the genome and therefore not considered in further analyses.

[8] Percentage of reads mapping to the genome and used for downstream analyses.

[9] Percentage of reads assigned to a feature after featureCounts analysis.

Supplementary Table 4. Blast search results for the top 5% highly expressed genes against the ncbi conserved domains database (CDD).

The protein sequences of the 5% most highly expressed genes (top5%) were used as query to Blast search the NCBI CDD database (https://www.ncbi.nlm.nih.gov/Structure/bwrpsb/bwrpsb.cgi).

Supplementary Table 4 is available as an additional file.

Supplementary Table 5. Members of known effector families in the top5% HE genes, in the genes DE between isolates and in the genes DE between *formae speciales*.

			HE genes[3]		DE between isolates[4]					DE between *formae speciales*[5]			
Avr family	Members total[1]	Members in *B.g. tritici*[2]	All top5%	HE core set	96224 vs 94202	JIW2 vs 94202	96224 vs JIW2	S-1391 vs S-1459	T3-8 vs THUN-12	*B.g. tritici* vs *B.g. secalis*	*B.g. tritici* vs *B.g. triticale*	*B.g. secalis* vs *B.g. triticale*	Other members[6]
BEC1016	78	33	14	-	1	4	3	0	1	17	3	14	
BEC1038	76	34	8	-	1	3	1	1	1	12	1	10	
AvrPm3[a2/f2]	46	30	15	4	3	3	5	3	5	10	2	8	
AvrPm2	26	14	7	1	1	2	1	-	-	4	1	5	Avr_{a13}, BEC1011, BEC1054
SvrPm3[a1/f1]	20	13	4	-	1	3	-	1	1	7	1	4	
CSEP0105	11	7	4	1	-	-	-	-	2	3	2	3	
BEC1018	7	3	1	1	1	1	-	-	-	2	2	-	
CSEP0055	6	2	-	-	-	-	-	-	-	1	-	1	
BEC1040	2	1	1	-	-	-	-	-	-	1	-	-	

[1] Total number of effector family members in *B.g. tritici* and *B.g. secalis*

[2] Number of effector family members in *B.g. tritici* only

[3] Number of effector family members present in the top 5% and in the core set of highly expressed genes.

[4] Number of effector family members corresponding to differentially expressed genes between isolates of the same *forma specialis*.

[5] Number of effector family members corresponding to differentially expressed genes *formae speciales*.

[6] Other functionally characterized members of the family.

Supplementary Table 6. Total number of genes and effector genes showing presence/absence polymorphisms between the reference isolate 96224 and the six other isolates used in this study.

	B.g. tritici		*B.g. secalis*		*B.g. triticale*	
Absent genes	JIW2	94202	S-1391	S-1459	T3-8	THUN-12
Genes	19	13	147	150	39	42
Effectors	4	3	72	73	16	15
Effectors (%)	21,1	23,1	49,0	48,7	41,0	35,7

Supplementary Table 7. Blast search results for the genes differentially expressed between isolates of the same *forma specialis* against the ncbi conserved domains database (CDD).

The protein sequences of the genes differentially expressed between isolates of the same *forma specialis* were used as query to Blast search the NCBI CDD database (https://www.ncbi.nlm.nih.gov/Structure/bwrpsb/bwrpsb.cgi).

Supplementary Table 7 is available as an additional file.

Supplementary Table 8: Comparison of the gene sets identified as differentially expressed with four different mapping strategies.

			Comparison between isolates[4]					Comparison between *formae speciales*[5]		
Mapping[1]	Number of mismatches[2]	Number of multimappers[3]	96224 vs 94202	JIW2 vs 94202	96224 vs JIW2	S-1391 vs S-1459	T3-8 vs THUN-12	*B.g. secalis* vs *B.g. tritici*	*B.g. secalis* vs *B.g. triticale*	*B.g. tritici* vs *B.g triticale*
1	0.01	1	68	93	69	46	69	335	245	68
2	0.04	1	65	92	69	46	70	314	230	66
3	0.01	20	69	95	69	49	73	363	272	70
4	0.04	20	64	90	68	44	72	318	234	69
Common[6]			62	85	65	40	62	291	216	58
Percentage of the genes identified with mapping 1 that were identified in all mappings[7]			91%	91%	94%	87%	90%	87%	88%	85%

[1] Mapping strategy

[2] Number of mismatches allowed per read

[3] Number of multimappers allowed

[4] Number of genes identified as differentially expressed between isolates of the same *forma specialis*.

[5] Number of genes identified as differentially expressed between *formae speciales*.

[6] Number of genes identified as differentially expressed with the four different mapping criteria.

[7] Percentage of genes identified in mapping 1 that are commonly found with all mapping strategies.

Supplementary Table 9. Blast search results for the genes differentially expressed between *formae speciales* against the ncbi conserved domains database (CDD).

The protein sequences of the genes differentially expressed between *formae speciales* were used as query to Blast search the NCBI CDD database (https://www.ncbi.nlm.nih.gov/Structure/bwrpsb/bwrpsb.cgi).

Supplementary Table 9 is available as an additional file.

Supplementary Table 10. Analysis of effector gene enrichment in DE genes using different thresholds for expression fold change differences.

	log2FC>|1|			log2FC>|1.5|			log2FC>|2|			log2FC>|2.5|		
Comparison	Genes	Effectors	Effectors(%)	Genes	Effectors	Effectors(%)	Genes	Effectors	Effectors(%)	Genes	Effectors	Effectors(%)
96224 vs 94202	140	34	24.3	62	22	35.5	31	13	41.9	21	7	33.3
JIW2 vs 94202	223	51	22.9	84	27	32.1	45	16	35.6	20	6	30.0
96224 vs JIW2	157	46	29.3	62	21	33.9	34	13	38.2	21	9	42.9
S-1391 vs S-1459	112	21	18.8	36	9	25.0	16	5	31.3	10	3	30.0
T3-8 vs THUN-12	106	48	45.3	57	27	47.4	37	20	54.1	25	13	52.0
B.g. tritici vs *B.g. secalis*	453	196	43.3	257	146	56.8	168	104	61.9	124	83	66.9
B.g. tritici vs B.g. triticale	164	75	45.7	58	35	60.3	31	20	64.5	22	14	63.6
B.g. secalis vs B.g. triticale	287	136	47.4	175	107	61.1	126	77	61.1	91	57	62.6

Supplementary Table 11. Comparison of gene composition in the most highly expressed genes (most HE genes), the genes differentially expressed between *formae speciales* (DE$^{ff.spp.}$) and the non parent-of-origin expressed genes.

	Total[1]	Effectors	Non-Effectors			
			Signal peptide	Secretory pathway (no signal peptide)	Mitochondrion	Ribosome
Most HE genes	418	112	13	20	55	90
DE $^{ff.spp.}$	292	166	7	9	16	2
Non parent-of-origin expressed genes	16	11	2	0	1	0

[1] The total number of genes in each category.

[2] The number of effector genes.

[3] The number of non-effector genes that have a signal peptide.

[4] The number of non-effector genes that don't have a signal peptide but are part of the secretory pathway.

[5] The number of genes that are part of the mitochondrion.

[6] The number of genes that are part of the ribosome.

We considered the secretome as genes in [1], [2] and [3].

3 Supplementary Texts

Supplementary Text 1. Genome coverage analysis to identify gene presence/absence polymorphism.

The aim of this analysis was to identify instances of gene presence/absence polymorphism based on sequence coverage. For this we used the reference genome sequence of the *B.g. tritici* isolate 96224, and Illumina re-sequencing data for the remaining six isolates used in this study (Wicker *et al.*, 2013; Menardo *et al.*, 2016). Here, the rpkm values obtained with genome sequencing reads are indicative of the actual presence or absence of the gene. We used the same pipeline that we employed for our RNAseq study to calculate gene sequence coverage using the same parameters as for gene expression studies. Illumina-Seq reads from the six non-reference isolates were mapped on the *B.g. tritici* reference genome (see Methods) using STAR (Dobin *et al.*, 2013) allowing one mismatch per 100 bp and no multi mappers. To obtain a read count value for sequence coverage of the genes we used featureCount 1.4.6 (Liao *et al.*, 2014). Normalizations were then performed on library size gene length similar to the RNAseq standards. Thus, a coverage value was obtained for every gene in every isolate and the distributions of those values were plotted as a function of the number of genes (Supplementary Figure 1). We used previously identified instances of gene polymorphism in the reference genome (Wicker et al., 2013; Praz et al., 2017) as a control to assess the minimum threshold for considering a gene as absent in an isolate compared to the reference. One prominent example is *AvrPm2*, which is present in the 96224 reference isolate but absent from the 94202 isolate. Based on manual inspection of coverage data, and the cited controls, we were able to set the threshold of 5x coverage as a cut off value for defining gene presence absence polymorphism. Thus, we obtained a list of genes that do not pass the critical 5x threshold which were therefore considered absent in the corresponding isolate (see Supplementary Table 4).
