# Supplementary material for: Non-parent of Origin Expression of Numerous Effector Genes Indicates a Role of Gene Regulation in Host Adaption of the Hybrid Triticale Powdery Mildew Pathogen
Source: Front Plant Sci. 2018 Jan 30;9:49. doi: 10.3389/fpls.2018.00049 (PMC5797619; doi:10.3389/fpls.2018.00049)
Supplement: Supplementary file 5 [file DataSheet1.DOCX]

##############################################

############# Load edgeR package #############

##############################################

library("edgeR")

##############################################

######## Read the featureCounts files ########

##############################################

f <- dir(,"bam_fc$")

x <- read.table(f[1],header=TRUE,sep="\t")

fs <- lapply(f, read.table, header=TRUE,sep="\t")

ds <- sapply(fs, function(u) u[,7])

### Add Geneid as row names

rownames(ds) <- x$Geneid

### Add and simplify column names

colnames(ds) <- gsub("Aligned.sortedByCoord.out.bam_fc","",f)

colnames(ds) <- gsub("_2dpi","",colnames(ds))

colnames(ds) <- gsub("_mat","",colnames(ds))

colnames(ds) <- gsub("_timbo","",colnames(ds))

### Create a DGEList object

d <- DGEList(ds)

### Calculate normalization factors to scale the raw library sizes

d <- calcNormFactors(d)

### Add information about the genes (Chromosome, Start, End, Strand and Length)

d$genes <- x[,1:6]

##############################################

### Set a threshold for minimal expression ###

##############################################

### A gene is consider expressed if there are at least 10 reads mapping on three replicates

k <- rowSums(cpm(d)>10) >= 3

### Select only genes that are expressed

d <- d[k,]

##############################################

########### Calculate RPKM values ###########

##############################################

### Calculate rpkm values

rpks <- rpkm(d, gene.length=d$genes$Length)

as.data.frame(rpks) -> rpks_df

### Add mean values for each isolates

(rpks_df[,1]+rpks_df[,2]+rpks_df[,3])/3 -> rpks_df$mean94

(rpks_df[,4]+rpks_df[,5]+rpks_df[,6])/3 -> rpks_df$mean96

(rpks_df[,7]+rpks_df[,8]+rpks_df[,9])/3 -> rpks_df$meanJI

(rpks_df[,10]+rpks_df[,11]+rpks_df[,12])/3 -> rpks_df$meanS1391

(rpks_df[,13]+rpks_df[,14]+rpks_df[,15])/3 -> rpks_df$meanS1459

(rpks_df[,16]+rpks_df[,17]+rpks_df[,18])/3 -> rpks_df$meanT38

(rpks_df[,19]+rpks_df[,20]+rpks_df[,21])/3 -> rpks_df$meanTHUN12

### Add mean values per forma specialis

(rpks_df$mean94 + rpks_df$meanJI + rpks_df$mean96)/3 -> rpks_df$meanBgt

(rpks_df$meanS1391 + rpks_df$meanS1459 )/2 -> rpks_df$meanBgs

(rpks_df$meanT38 + rpks_df$meanTHUN12 )/2 -> rpks_df$meanBgtriticale

### Simplify rownames

rownames(rpks_df) <- gsub(".path1","",rownames(rpks_df))

##############################################

####### Multidimensional scaling plot #######

##############################################

plotMDS(d)

##############################################

########## Highly expressed genes ###########

##############################################

### 6203 genes are expressed

nrow(rpks_df)

### The top10% are the 620 genes the most highly expressed

### The top5% are the 310 genes the most highly expressed

### Select top5% and top10% for each isolate

head(rpks_df[order(-rpks_df$mean96),], n=310) -> top5percent_96

head(rpks_df[order(-rpks_df$mean96),], n=620) -> top10percent_96

head(rpks_df[order(-rpks_df$mean94),], n=310) -> top5percent_94

head(rpks_df[order(-rpks_df$mean94),], n=620) -> top10percent_94

head(rpks_df[order(-rpks_df$meanJI),], n=310) -> top5percent_JI

head(rpks_df[order(-rpks_df$meanJI),], n=620) -> top10percent_JI

head(rpks_df[order(-rpks_df$meanS1391),], n=310) -> top5percent_S1391

head(rpks_df[order(-rpks_df$meanS1391),], n=620) -> top10percent_S1391

head(rpks_df[order(-rpks_df$meanS1459),], n=310) -> top5percent_S1459

head(rpks_df[order(-rpks_df$meanS1459),], n=620) -> top10percent_S1459

head(rpks_df[order(-rpks_df$meanT38),], n=310) -> top5percent_T38

head(rpks_df[order(-rpks_df$meanT38),], n=620) -> top10percent_T38

head(rpks_df[order(-rpks_df$meanTHUN12),], n=310) -> top5percent_THUN12

head(rpks_df[order(-rpks_df$meanTHUN12),], n=620) -> top10percent_THUN12

### Look for effector enrichment

##############################################

############## DE analyses 1 #################

######## Comparison between isolates #########

##############################################

### Define one group per isolate

group <- gsub("_[123]","",colnames(ds))

### Create a design matrix

design_iso <- model.matrix(~group)

### Estimate common negative binomial dispersion parameter

d <- estimateGLMCommonDisp(d,design_iso)

### Estimate abundance-dispersion trend by Cox-Reid approximate profile likelihood

d <- estimateGLMTrendedDisp(d,design_iso)

### Estimate empirical Bayes estimate of the negative binomial dispersion parameter for each tag

d <- estimateGLMTagwiseDisp(d,design_iso)

### Fit a negative binomial generalized log-linear model to the read counts for each gene

f <- glmFit(d,design_iso)

### Conduct likelihood ratio tests

lrt1 <- glmLRT(f,coef=2) # 96224 vs 94202

lrt2 <- glmLRT(f,coef=3) # JIW2 vs 94202

lrt3 <- glmLRT(f,contrast=c(0,-1,1,0,0,0,0)) # 96224 vs JIW2

lrtBgs <- glmLRT(f,contrast=c(0,0,0,-1,1,0,0)) # S-1391 vs S-1459

lrtBgtriticale <- glmLRT(f,contrast=c(0,0,0,0,0,-1,1)) # T3-8 vs THUN-12

##############################################

###### Select genes DE between isolates ######

######## of the same forma speciales #########

##############################################

### logFC > 1.5, FDR < 0.01

topTags(lrt1, n="infinite") -> lrt1_tt

lrt1_tt$table -> lrt1_tt_tab

lrt1_tt_tab[(abs(lrt1_tt_tab$logFC) > 1.5) & (lrt1_tt_tab$FDR < 0.01),] -> lrt1_tt_DE

nrow(lrt1_tt_DE)

topTags(lrt2, n="infinite") -> lrt2_tt

lrt2_tt$table -> lrt2_tt_tab

lrt2_tt_tab[(abs(lrt2_tt_tab$logFC) > 1.5) & (lrt2_tt_tab$FDR < 0.01),] -> lrt2_tt_DE

nrow(lrt2_tt_DE)

topTags(lrt3, n="infinite") -> lrt3_tt

lrt3_tt$table -> lrt3_tt_tab

lrt3_tt_tab[(abs(lrt3_tt_tab$logFC) > 1.5) & (lrt3_tt_tab$FDR < 0.01),] -> lrt3_tt_DE

nrow(lrt3_tt_DE)

topTags(lrtBgs, n="infinite") -> lrtBgs_tt

lrtBgs_tt$table -> lrtBgs_tt_tab

lrtBgs_tt_tab[(abs(lrtBgs_tt_tab$logFC) > 1.5) & (lrtBgs_tt_tab$FDR < 0.01),] -> lrtBgs_tt_DE

nrow(lrtBgs_tt_DE)

topTags(lrtBgtriticale, n="infinite") -> lrtBgtriticale_tt

lrtBgtriticale_tt$table -> lrtBgtriticale_tt_tab

lrtBgtriticale_tt_tab[(abs(lrtBgtriticale_tt_tab$logFC) > 1.5) & (lrtBgtriticale_tt_tab$FDR < 0.01),] -> lrtBgtriticale_tt_DE

nrow(lrtBgtriticale_tt_DE)

### For further analyses, remove genes that are absent in one of the isolates

### Look for effector enrichment

##############################################

############## DE analyses 2 #################

#### Comparison between formae speciales #####

##############################################

### Create a design matrix

targets_fsp <- read.table("targets_fsp", header=TRUE)

group_fsp <- targets_fsp$formaspecialis

design_fsp <- model.matrix(~group_fsp)

### Estimate common negative binomial dispersion parameter

d <- estimateGLMCommonDisp(d,design_fsp)

### Estimate abundance-dispersion trend by Cox-Reid approximate profile likelihood

d <- estimateGLMTrendedDisp(d,design_fsp)

### Estimate empirical Bayes estimate of the negative binomial dispersion parameter for each tag

d <- estimateGLMTagwiseDisp(d,design_fsp)

### Fit a negative binomial generalized log-linear model to the read counts for each gene

f <- glmFit(d,design_fsp)

### Conduct likelihood ratio tests

lrtST <- glmLRT(f,coef=2) # B.g. secalis vs B.g. tritici

lrtSTR <- glmLRT(f,coef=3) # B.g. secalis vs B.g. triticale

lrtTTR <- glmLRT(f,contrast=c(0,-1,1)) # B.g. tritici vs B.g. triticale

##############################################

## Select genes DE between formae speciales ##

##############################################

### logFC > 1.5, FDR < 0.01

topTags(lrtST, n="infinite") -> lrtST_tt

lrtST_tt$table -> lrtST_tt_tab

lrtST_tt_tab[(abs(lrtST_tt_tab$logFC) > 1.5) & (lrtST_tt_tab$FDR < 0.01),] -> lrtST_tt_DE

nrow(lrtST_tt_DE)

topTags(lrtSTR, n="infinite") -> lrtSTR_tt

lrtSTR_tt$table -> lrtSTR_tt_tab

lrtSTR_tt_tab[(abs(lrtSTR_tt_tab$logFC) > 1.5) & (lrtSTR_tt_tab$FDR < 0.01),] -> lrtSTR_tt_DE

nrow(lrtSTR_tt_DE)

topTags(lrtTTR, n="infinite") -> lrtTTR_tt

lrtTTR_tt$table -> lrtTTR_tt_tab

lrtTTR_tt_tab[(abs(lrtTTR_tt_tab$logFC) > 1.5) & (lrtTTR_tt_tab$FDR < 0.01),] -> lrtTTR_tt_DE

nrow(lrtTTR_tt_DE)

### For further analyses, remove genes that are absent in one of the formae speciales

### Look for effector enrichment

##############################################

############## DE analyses 3 #################

######### Expression in the hybrid ###########

##############################################

### Create a design matrix

targets_hybrid <- read.table("targets_hybrid", header=TRUE)

group_hybrid <- targets_hybrid$hybrid

design_hybrid <- model.matrix(~group_hybrid)

### Estimate common negative binomial dispersion parameter

d <- estimateGLMCommonDisp(d,design_hybrid)

### Estimate abundance-dispersion trend by Cox-Reid approximate profile likelihood

d <- estimateGLMTrendedDisp(d,design_hybrid)

### Estimate empirical Bayes estimate of the negative binomial dispersion parameter for each tag

d <- estimateGLMTagwiseDisp(d,design_hybrid)

### Fit a negative binomial generalized log-linear model to the read counts for each gene

f <- glmFit(d,design_hybrid)

### Conduct likelihood ratio tests

lrtHyb_TriT38 <- glmLRT(f,contrast=c(0,-1,1,0)) # T3-8 vs B.g. tritici

lrtHyb_TriTHUN12 <- glmLRT(f,contrast=c(0,-1,0,1)) # THUN-12 vs B.g. tritici

lrtHyb_SecT38 <- glmLRT(f,coef=3) # T3-8 vs B.g. secalis

lrtHyb_SecTHUN12 <- glmLRT(f,coef=4) # THUN-12 vs B.g. secalis

##############################################

######### Select genes DE between one ########

### B.g.triticale isolate and B.g. tritici ###

##############################################

topTags(lrtHyb_TriT38, n="infinite") -> lrtHyb_TriT38_tt

lrtHyb_TriT38_tt$table -> lrtHyb_TriT38_tt_tab

lrtHyb_TriT38_tt_tab[(abs(lrtHyb_TriT38_tt_tab$logFC) > 1.5) & (lrtHyb_TriT38_tt_tab$FDR < 0.01),] -> lrtHyb_TriT38_tt_DE

topTags(lrtHyb_TriTHUN12, n="infinite") -> lrtHyb_TriTHUN12_tt

lrtHyb_TriTHUN12_tt$table -> lrtHyb_TriTHUN12_tt_tab

lrtHyb_TriTHUN12_tt_tab[(abs(lrtHyb_TriTHUN12_tt_tab$logFC) > 1.5) & (lrtHyb_TriTHUN12_tt_tab$FDR < 0.01),] -> lrtHyb_TriTHUN12_tt_DE

### For further analyses, remove genes that are absent

##############################################

######### Select genes DE between one ########

### B.g.triticale isolate and B.g. secalis ###

##############################################

topTags(lrtHyb_SecT38, n="infinite") -> lrtHyb_SecT38_tt

lrtHyb_SecT38_tt$table -> lrtHyb_SecT38_tt_tab

lrtHyb_SecT38_tt_tab[(abs(lrtHyb_SecT38_tt_tab$logFC) > 1.5) & (lrtHyb_SecT38_tt_tab$FDR < 0.01),] -> lrtHyb_SecT38_tt_DE

topTags(lrtHyb_SecTHUN12, n="infinite") -> lrtHyb_SecTHUN12_tt

lrtHyb_SecTHUN12_tt$table -> lrtHyb_SecTHUN12_tt_tab

lrtHyb_SecTHUN12_tt_tab[(abs(lrtHyb_SecTHUN12_tt_tab$logFC) > 1.5) & (lrtHyb_SecTHUN12_tt_tab$FDR < 0.01),] -> lrtHyb_SecTHUN12_tt_DE

### For further analyses, remove genes that are absent

##############################################

####### Workflow for further analyses ########

##############################################

# Find the genes for which expression in T3-8 = Bgs (abs(logFC) < 1.5)

# Find the genes for which expression in T3-8 = Bgt

# Find the genes for which expression in THUN-12 = Bgs

# Find the genes for which expression in THUN-12 = Bgt

# Remove genes which expression is similar to both Bgt and Bgs

# Compare expression level (Bgs or Bgt) with genomic origin of the gene (Bgs or Bgt)

# Identify genes that have non-parent-of-origin expression level

### Look for effector enrichment
